# Greener Synthesis of the Polymer of Intrinsic Microporosity PIM-1 for Gas Separation

**DOI:** 10.1021/acssuschemeng.4c08475

**Published:** 2024-12-31

**Authors:** Alisha Ayyaz, Andrew B. Foster, Levente Cseri, Gyorgy Szekely, Peter M. Budd

**Affiliations:** † Department of Chemistry, The University of Manchester, Manchester M13 9PL, United Kingdom; ‡ Department of Chemical Engineering, The University of Manchester, Manchester M13 9PL, United Kingdom; § Advanced Membranes and Porous Materials Center, Physical Science and Engineering Division (PSE), King Abdullah University of Science and Technology (KAUST), Thuwal 23955-6900, Saudi Arabia

**Keywords:** green solvent, PIM-1, PIM, polymers of intrinsic microporosity, green metrics analysis, gas separation, membranes, green synthesis

## Abstract

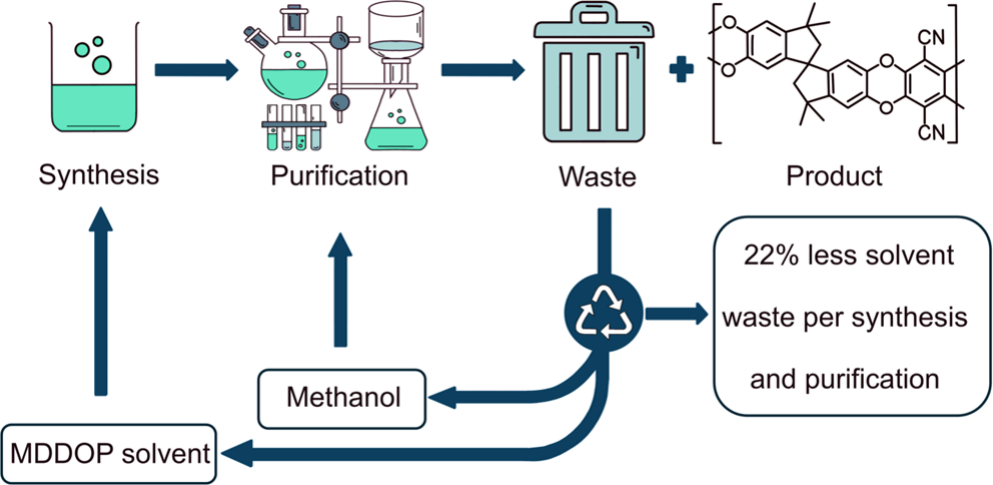

Polymers of intrinsic microporosity (PIMs) are studied as membranes for energy-efficient and environmentally friendly separation technologies, but greener polymerization methods are desirable for further scale up. This work aimed to synthesize the prototypical PIM (PIM-1) via a greener synthetic route by changing the solvent system to methyl-5-(dimethylamino)-2,2-dimethyl-5-oxopentanoate (MDDOP), a structural analogue of the green solvent Rhodiasolv PolarClean. Mass-based green metrics analysis was performed on MDDOP, determining atom economy, complete environmental factor, and total carbon intensity, comparing each to synthetic routes to PolarClean. Green metrics analysis found MDDOP synthesis produced less waste than PolarClean. MDDOP solvent capabilities were exemplified via PIM-1 polymerizations using 5,5′,6,6′-tetrahydroxy-3,3,3′,3′-tetramethyl-1,1′-spirobisindane (TTSBI) with either tetrafluoroterephthalonitrile (TFTPN) or tetrachloroterephthalonitrile (TCTPN), varying the temperature (120–160 °C) and reaction duration (50 min–6 h). Recovery of methanol and MDDOP post PIM-1 synthesis reduced solvent waste by 22%. Reactions using TCTPN produced polymers with higher molar masses than those produced using TFTPN. All samples showed varied topology, with evidence of branching and colloidal network. The polymer from the most successful reaction conditions (TCTPN, *T* = 140 °C, 6 h) was fabricated into thick film membranes and tested with pure gases for CO_2_/CH_4_ and CO_2_/N_2_ gas pairs, performing comparably with PIM-1 synthesized using conventional solvent systems.

## Abstract 2

Hazardous solvents used in PIM-1 synthesis were replaced with a potential green solvent (MDDOP), which was recovered after synthesis, reducing solvent waste.

## 1 Introduction

The impact of global warming has become apparent in recent years, with CO_2_ emissions reaching an all-time high of 37.4 Gt in 2023,^1^ making the ongoing effort to achieve the sustainable development goals (SDGs) by 2030 crucial.^2^ Polymers of intrinsic microporosity (PIMs) can be used as membranes for gas and liquid separations. Therefore, they could play an integral role in the shift to more sustainable and less energy-intensive processes.^3^

PIMs are a group of amorphous polymers that exhibit intrinsic microporosity (IM).^3−5^ Their IM arises from a network of interconnected free volume elements, due to their rigid and contorted polymer backbones.^4^ The archetypal PIM is PIM-1.^3^ PIM-1 is synthesized via step-growth polymerization of 5,5′,6,6′-tetrahydroxy-3,3,3′,3′-tetramethyl-1,1′-spirobisindane (TTSBI) with either tetrafluoroterephthalonitrile (TFTPN) or tetrachloroterephthalonitrile (TCTPN).^3^ Currently, PIM-1 is commonly synthesized using TTSBI and TFTPN, in a solvent system of DMAc and toluene (2:1) at 160 °C for ∼1 h, with additions of solvent at regular intervals throughout.^6^ The structural composition of PIM-1 varies depending on its polymerization conditions. By modifying reaction conditions, branched, cyclic, tadpole, and network topologies can be introduced.^7,8^ These variations were exemplified by replacing TFTPN with TCTPN, its cheaper, less reactive equivalent. TCTPN also has a lower global warming potential and toxicity than TFTPN.^9^

PIM membranes can suffer the effects of physical aging. To prolong the lifespan of PIM-1 membranes, alcohol treatments can be utilized. On a laboratory scale, soaking PIM-1 films in lower alcohols, e.g., methanol and ethanol, can reverse the reduction in permeability due to physical aging.^10^ In an industrial setting, regeneration could be achieved by treatment with methanol vapor, for both self-standing and thin film composite membranes.^10,11^

The 12 principles of green chemistry serve as a guide when altering existing or developing new methods and materials, helping to reduce their negative environmental impact.^12,13^ The first principle of green chemistry suggests that processes should be designed with waste prevention in mind.^13^ Mass-based green metrics analysis determines the wastefulness of production with respect to the mass of reagents and the generated waste. The fifth principle of green chemistry states that solvents should be avoided if possible, but where unavoidable, they must be nontoxic and harmless.^12^ Unfortunately, some of the most commonly used solvents are extremely toxic and hazardous.^14^ The solvents currently used for PIM-1 synthesis, i.e., toluene and DMAc, are classed as problematic and hazardous, respectively, by the Innovative Medicines Initiative (IMI)–CHEM 21.^14^ For greener PIM-1 synthesis, greener solvent alternatives must be used and waste reduction must be implemented. Polar aprotic solvents have few green alternatives, among which methyl 5-(dimethylamino)-2-methyl-5-oxopentanoate (PolarClean) is popular. In 2019, Cseri and Szekely^15^ aimed to improve the synthesis of PolarClean, as it is a mixture of compounds. They also synthesized methyl 5-(dimethylamino)-2,2-dimethyl-5-oxopentanoate (MDDOP), a structural analogue of PolarClean thought to have similar properties.^15^

The present work aimed to assess MDDOP as a green solvent and use it for PIM-1 synthesis. This assessment required the green metrics analysis of MDDOP, calculating atom economy, complete environmental factor, and total carbon intensity. These values were compared to its commercially available, structural analogue PolarClean. The solvent capabilities of MDDOP are demonstrated by reaction optimization of PIM-1 polymerization. Pure gas separation for CO_2_/CH_4_ and CO_2_/N_2_ gas pairs, from self-standing membranes prepared from PIM-1 produced using MDDOP, performed comparably with conventionally synthesized PIM-1.

## 2 Experimental Section

### 2.1 MDDOP Synthesis and Isolation

A two-neck round-bottom flask was fitted with an Ar inlet and purged. To this, 25 g (0.245 mol, 28 mL) of methyl isobutyrate and 22.05 g (0.222 mol, 22.9 mL) of dimethylacrylamide (DMAA) were added and stirred. The mixture was placed in an ice bath. Potassium tert-butoxide (835 mg) was added slowly to the mixture over 30 min and stirred for a further 2 min before quenching. The reaction was quenched by slowly adding 12.5 mL of oxalic acid (1 M) solution. The solution was allowed to warm to room temperature, and the solvent was isolated via reduced pressure distillation (temperature and vacuum pressure are in Supporting Information).

### 2.2 MDDOP Mass-Based Green Metrics Analysis

Mass-based green metrics analysis calculations for atom economy (AE), complete environmental factor (cEF) and total carbon intensity (CI_total_), can be used for quantification of waste production.^15^ AE, cEF, and CI_total_ were calculated for the synthesis and isolation of MDDOP using eqs 1–6.
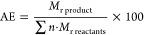
1where *M*_r product_ and *M*_r reactants_ are the molecular masses of the desired product and reactants, respectively.
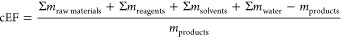
2where ∑*m*_raw materials_, ∑*m*_reagents_, ∑*m*_solvents_, and ∑*m*_water_ are the total masses of raw materials, reagents, solvents and water used, respectively, for all synthesis steps and *m*_products_ is the mass of desired products.
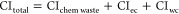
3where CI_chem waste_ is the carbon intensity of chemical waste, CI_ec_ is the energy consumption, and CI_wc_ cooling water consumption.
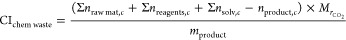
4where *n*_raw mat,c_, *n*_reagents,c_, *n*_solv,c_, and *n*_product,c_ are the amounts of carbon in the raw materials, reagents, solvents, and synthesized product, respectively,  is the molecular mass of CO_2_, and *m*_product_ is the mass of product. CI_ec_ is expressed as eq 5.
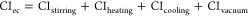
5where CI_stirring_, CI_heating_, CI_cooling_ and CI_vacuum_ are the carbon intensities from the total amount of stirring, heating, cooling and vacuum used to produce 100 g of the final product. CI_wc_ is expressed as eq 6.
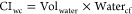
6where Vol_water_ is the volume of water required (m^3^) and Water_cf_ is a conversion factor that converts the amount of water used to the amount of CO_2_ produced for the said water production.

### 2.3 PIM-1 Synthesis and Purification

A series of polymerizations were performed, as summarized in Table 1. Equimolar amounts of TTSBI (3.460 g, 10 mmol) and TFTPN or TCTPN (2.00 or 2.708 g, 10 mmol), potassium carbonate (4.146 g, 30 mmol), and 30 mL of MDDOP were added to a 100 mL three-neck, round-bottom flask. The mixture was heated from room temperature to the desired reaction temperature with continuous stirring using a magnetic stirrer bar and a DrySyn aluminum heating block fitted with a temperature probe. Reaction conditions investigated in this work are listed in Table 1.

**Table 1:** Reaction Conditions for PIM-1 Synthesis Using MDDOP

reaction conditions			
monomer	temperature (°C)	time (h)	polymer produced
TFTPN	140	0.83	F1
TFTPN	140	6	F2
TFTPN	160	2	F3
TCTPN	120	6	C1
TCTPN	140	6	C2
TCTPN	160	2	C3

The reaction was quenched into excess methanol, which caused PIM-1 to precipitate. The polymer was isolated using vacuum filtration, and the filtered solution was collected. Methanol and MDDOP were recovered from the collected solution via rotary evaporation and reduced pressure distillation, respectively.

The recovered polymer was dissolved in chloroform (ca. 5 g/110 mL) and reprecipitated back in methanol (five times excess). The polymer was again isolated using a sintered funnel under vacuum. The dried polymer was added to 1 L of deionized water and refluxed at 90 °C for 16 h. The polymer was filtered again using a sintered funnel under vacuum and washed with a small amount of acetone. The filtered polymer was left to soak in methanol (100 mL) for 12 h and filtered again. Purified PIM-1 was dried in a vacuum oven at 120 °C for 2 days.

### 2.4 Polymer Characterization

Gel permeation chromatography (GPC) was used to determine the weight-average molar mass (*M*_w_), number-average molar mass (*M*_n_), and dispersity (*Đ* = *M*_w_/*M*_n_) of the polymers, via OmniSec software. GPC was carried out using a Viscotek VE2001 SEC solvent/sample module with two PL Mixed B columns, with Viscotek TDA 302 triple detector array (refractive index, light scattering, and viscosity detectors), on polymer solutions in chloroform (1 mg mL^–1^), which had been prefiltered through a 0.45 μm syringe filter. The injection volume was 100 μL, with a flow rate of 1 mL min^–1^.

^1^H NMR was performed using a Bruker AVANCE II 500 MHz instrument on polymer solutions (20 mg mL^–1^) in CDCl_3_.

A Shimadzu Biotech Axima Confidence instrument was used for matrix-assisted laser desorption ionization time-of-flight (MALDI-TOF) mass spectrometry analysis. Each PIM-1 sample (5 mg) was dissolved in chloroform (100 μL) and mixed with a matrix solution (dithranol in THF, 10 mg mL^–1^) in a 1:10 ratio (sample:matrix). This mixture was spotted onto a plate with sodium iodide solution (10 mg mL^–1^) using the “layered method”. Calibration was carried out using a spherical peptide mix (1600–3500 Da). Samples were run using the linear mode with the pulse extraction optimized to 7000 kg mol^–1^.

The amount of network present in each polymer was determined from a 1 mg mL^–1^ solution prepared using chloroform and 10 mg of polymer. The polymer was left to dissolve for 24 h. The solution was passed through a 0.45 μm syringe filter, with the collected mass of the filtered contents noted. The filtered solution was allowed to evaporate and placed in an oven until dryness. The sample was weighed again to determine the amount of recovered soluble PIM-1. The network content was determined by comparing the initial and final polymer concentrations.

Dynamic light scattering (DLS) was performed on a 50 ppm solution of PIM-1 in chloroform at 25 °C, using a Malvern Zetasizer Nano ZS instrument.

Elemental analysis was performed on all samples. Five mg of each polymer was tested for C, H, and N contents using a Flash 2000 Organic Elemental Analyzer.

Nitrogen adsorption and desorption isotherms were measured using a Micromeritics ASAP 2020 physisorption analyzer. The sample (100 mg) was degassed at 120 °C under vacuum (0.1 mmHg) for 16 h before the physisorption analysis at the liquid nitrogen temperature of −196 °C (77 K). The apparent specific surface area of the materials was determined from the adsorption isotherm data, in the relative pressure range from 0.03 to 0.3, using the Brunauer–Emmett–Teller (BET) method.

### 2.5 Self-Standing Membrane Fabrication

Self-standing membranes of the highest molar mass polymer C2 (described in Table 1) were cast in a poly(tetrafluoroethylene) (PTFE) Petri dish from 3% w/v solutions in chloroform (0.3 g polymer in 10 mL CHCl_3_). The solutions were left to dry for 4 days at room temperature in a positive pressure nitrogen atmosphere cabinet. The films were placed in a vacuum oven overnight at 100 °C to complete drying. Film thicknesses were recorded for all the samples (measured with a Mitutoyo digimatic micrometer).

### 2.6 Pure Gas Permeation Testing

Pure gas permeation testing was performed using N_2_, CH_4_, and CO_2_ at ∼298 K and gauge gas pressures of 35 psi (2.41 bar) for N_2_ and CH_4_ and 25 psi (1.72 bar) for CO_2_. The tubing was purged with N_2_ gas for 20 min to remove any residual moisture before testing.^16^

A coupon was cut from each thick film membrane with an effective area of 2.84 cm^2^ and tested with N_2_, CH_4_, and CO_2_. The coupons were placed into the permeation cell, and a rubber ring seal was placed atop them. Before each gas measurement, the gas pressure was set to 35 or 25 psi and left to purge for 5 min. At least six measurements were taken per coupon for each gas and averaged to obtain the final result, which was then used to calculate the gas permeance (eq 7).
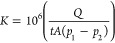
7where *K* is the permeance in gas permeation units (1 GPU = 10^–6^ cm^3^(STP) cm^–2^ s^–1^ cmHg^–1^ = 3.348 × 10^–10^ mol m^–2^ s^–1^ Pa^–1^). *Q* is the volume (cm^3^) of the permeated gas at STP (0 °C and 1 atm) through the membrane with an area of *A* (cm^2^) at time *t* (s). *p*_1_ and *p*_2_ are the feed and permeate side pressures (cmHg), respectively. Permeability (*P*) is calculated using eq 8.
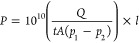
8where *P* is the permeability in Barrer (1 Barrer = 10^–10^ cm^3^(STP) cm^–2^ s^–1^ cmHg^–1^ = 3.348 × 10^–16^ mol m^–2^ s^–1^ Pa^–1^), and *l* is the membrane thickness (cm).

## 3 Results and Discussion

The MDDOP solvent was analyzed via green metrics analysis to determine the amount of waste generated during its synthesis and isolation. MDDOP was also used in several PIM-1 polymerizations to determine the optimum conditions for PIM-1 synthesis. Methanol and MDDOP were isolated from the waste streams of the quenching process via rotary evaporation and reduced pressure distillation, respectively. Recovered methanol was characterized by NMR and recovered MDDOP was characterized by gas chromatography–mass spectrometry (GCMS) and NMR. Each polymer sample was characterized via GPC, ^1^H NMR, MALDI-TOF, network content analysis, DLS and elemental analysis. The polymer recovered from the most successful reaction conditions (TCTPN, *T* = 140 °C, 6 h) was fabricated into thick film membranes and tested with single gases for the separations CO_2_/N_2_ and CO_2_/CH_4_.

### 3.1 Mass-Based Green Metrics Analysis

**Figure 1:**
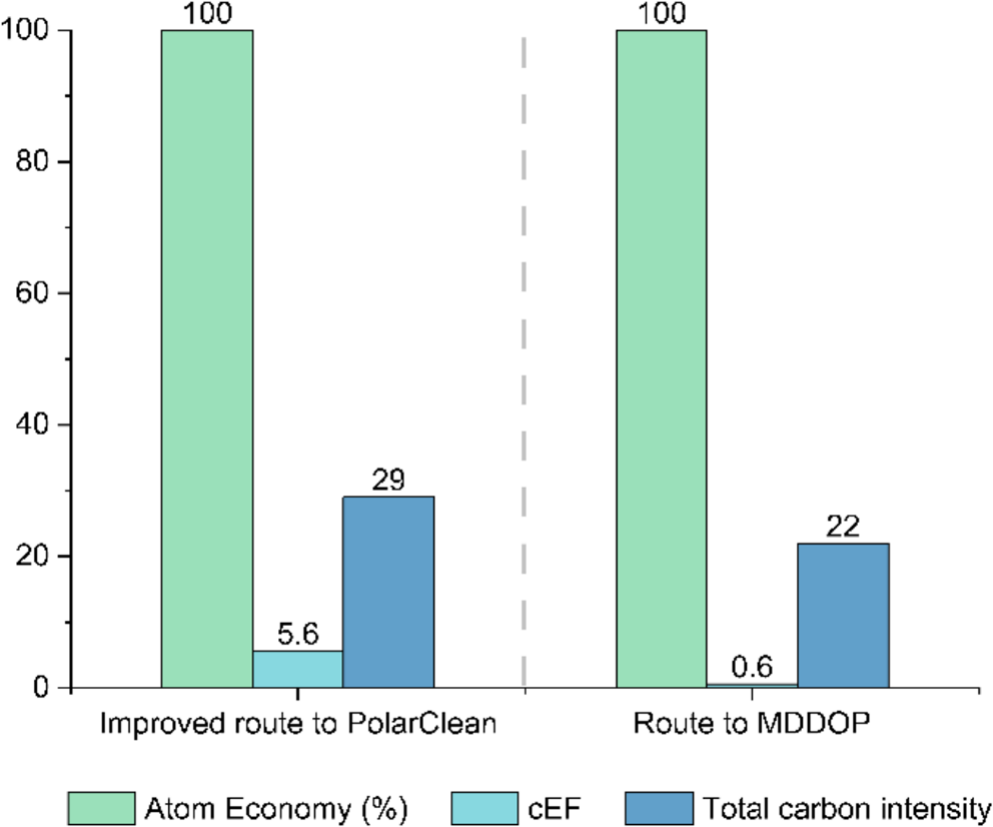
Cumulative atom economy percentage, complete environmental factor (cEF), and total carbon intensity (CI_total_) of improved synthetic route to PolarClean^15^ and route to MDDOP.

Mass-based green metrics analysis was performed on MDDOP. Methods outlined by Cseri and Szekely^15^ were followed to enable the direct comparison of MDDOP synthesis with the known routes for PolarClean synthesis. Their study aimed to improve the route to PolarClean by analyzing patented routes to PolarClean synthesis and their proposed new synthetic routes.^15^ Herein, the synthesis of MDDOP will be discussed and compared with the synthetic route to PolarClean that saw the greatest improvement (Route C in ref (15)). The AE, cEF, and CI_total_ were calculated. Figure 1 compares the results for MDDOP with the improved route to PolarClean.^15^

**Scheme 1:**
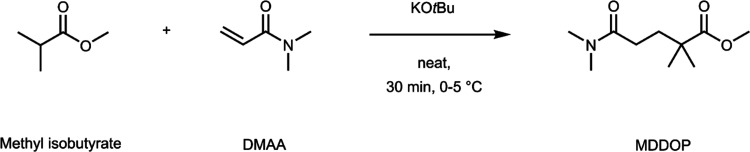
Reaction Scheme for MDDOP Synthesis

Figure 1 shows AE for the improved route to PolarClean and the route to MDDOP. AE quantifies the amount of reagents which appear in the desired product of a synthesis, assuming exact stoichiometric quantities and a chemical yield of 100%. AE can also help to approximate the amount of waste a synthetic step can produce; a smaller AE will generate higher amounts of waste materials. Both the improved route to PolarClean and the route to MDDOP have an atom economy of 100%, as seen in Figure 1. Scheme 1 shows the reaction scheme for the synthesis of MDDOP.

Methyl isobutyrate and DMAA undergo Michael addition to directly form MDDOP, without the need for additional steps to give the desired chemical structure.

Unlike AE, cEF accounts for the actual waste produced from a process by considering all auxiliary components and chemical yields,^17^ providing a comprehensive view of the waste associated with chemical production. The smaller the cEF, the lower the negative impact on the environment.^17^ A lower cEF may also indicate a lower manufacturing cost because of reduced amounts of hazardous material for disposal, which usually adds additional costs to chemical production.^18^ The route to MDDOP has the smallest cEF of 0.6%. Under solvent-free reaction conditions, waste is only generated from reaction quenching using the oxalic acid solution and the small amount of unreacted reagents. The improved route to PolarClean had a yield of 48%, whereas the route to MDDOP had a yield of 78%. This difference causes the large reduction in cEF for the route to MDDOP. A smaller cEF implies that the production of MDDOP would produce less waste than that of PolarClean, also reducing the cost of waste disposal. Less starting materials are required to make the same amount of MDDOP, reducing the cost further.

The CI_total_ of MDDOP was calculated in relation to the production of 100 g of MDDOP, using eq 3. CI_total_ is used to determine the mass emission of CO_2_ associated with the production of a unit mass of the given product.^15^ The lower the CI_total_, the less CO_2_ will be released. The CI_total_ of MDDOP was lower than that of the improved route to PolarClean, implying that less CO_2_ will be released during the synthesis of 100 g of MDDOP than that of PolarClean. The synthesis of MDDOP produces less CO_2_, therefore it would have a lower cost associated with CO_2_ disposal than the improved route to PolarClean. This calculation includes energy requirements for all aspects of the reaction process, such as chilling or pressure reduction.^15^

**Figure 2:**
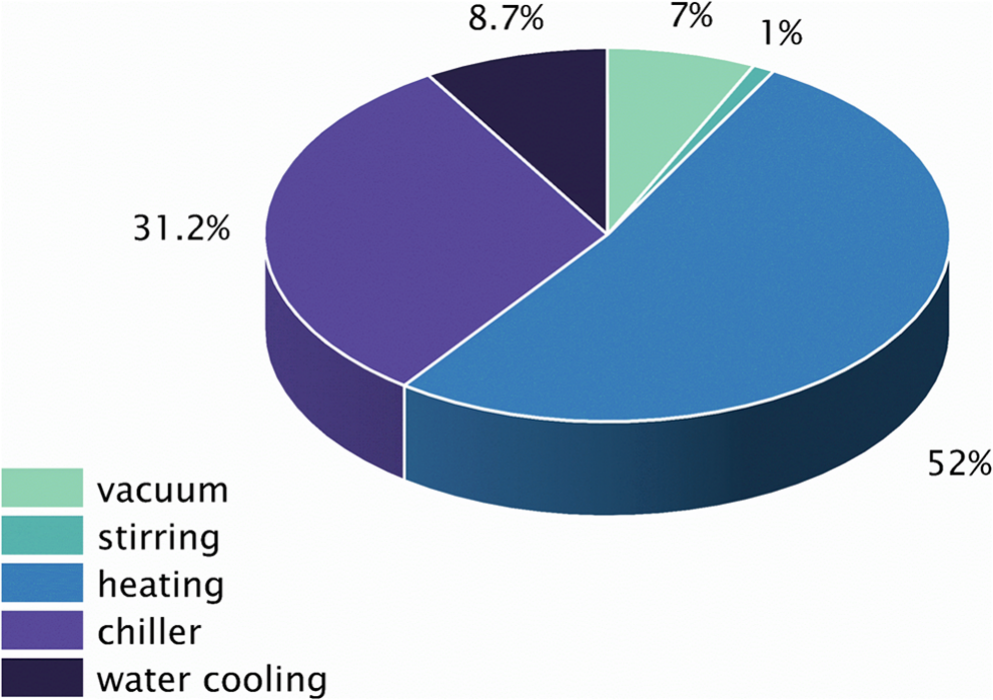
Breakdown of contributors to CI_ec_ to produce MDDOP.

Figure 2 shows a breakdown of the contributors to CI_ec_. Over half of the CO_2_ released during MDDOP synthesis was produced from heating during solvent isolation via reduced pressure distillation. The next largest contributor was chilling required during synthesis. Less than 20% of the CO_2_ produced resulted from water cooling and vacuum during distillation and stirring during synthesis and isolation.

### 3.2 PIM-1 Synthesis Using MDDOP

Six polymerizations of PIM-1 were performed using MDDOP, and five products were isolated in relatively low yields (84–90%). Such low yields indicate that the polymer contains significant amounts of branched structures, which excessively consume TTSBI within the step-growth polymerization. The product synthesized at the lowest polymerization temperature of 120 °C (C1) could not be recovered after extensive purification steps. This indicates that this product had lower molar mass than other isolated products. The different molar masses and degrees of branching of the five isolated products are shown in Table 2.

PIM-1 polymerizations performed in a single polar aprotic solvent or as part of a mixture are typically described as heterogeneous; the polymer does not remain fully dissolved in the solvent throughout the reaction, which is also the case with MDDOP. A relatively small number of polar aprotic solvents have been used successfully, typically *N*,*N*-dimethylformamide (DMF), DMAc and NMP, and more structural modeling work is required to better understand the role of the polarity of the solvent used in the polymerization.

### 3.3 Solvent Recovery

**Figure 3:**
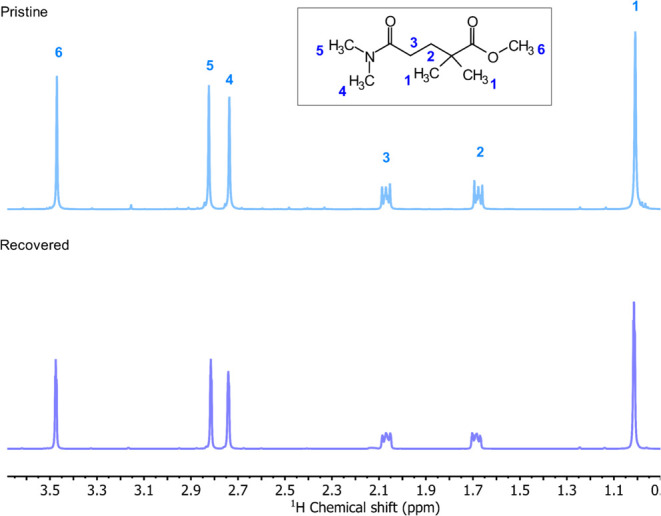
^1^H NMR of pure MDDOP before use in PIM-1 polymerization (top) and recovered MDDOP (bottom).

PIM-1 polymerizations were quenched using excess methanol before further purification, causing its precipitation, and facilitating its separation from the solvent waste. The waste stream contained a mixture of methanol, unreacted monomer, small oligomers, salt, base and, in this case, MDDOP. By isolating this solvent waste, methanol and MDDOP can be recovered. Rotary evaporation was used to recover methanol, thereby isolating 92% of this solvent. MDDOP has a high boiling point of 283.4 °C;^15^ therefore, reduced pressure distillation was used to isolate this solvent and 54% of MDDOP (>99% purity, GCMS) was recovered. By isolating these two solvents, the overall solvent waste of a PIM-1 synthesis and purification (polymer C2 reaction conditions) was reduced by 22%. Figure 3 shows the ^1^H NMR spectra of MDDOP before and after isolation.

A comparison of the two spectra shows that the MDDOP structure remained unchanged after being used in polymerization, followed by isolation from waste streams. MDDOP can therefore be regarded as stable under the reaction conditions of PIM-1 synthesis and can be recycled for further use.

### 3.4 Polymer Characterization

#### 3.4.1 GPC Analysis Including Mark–Houwink Plots

PIM-1 is a step-growth polymer obtained via nucleophilic aromatic substitution (S_N_Ar) reactions between TTSBI and TXTPN monomers, where X = F or C (C=Cl). S_N_Ar reactions proceed via attack of a nucleophile at an electron deficient aromatic carbon, attached to a leaving group; this step is also the rate determining step.^20^ The more electronegative the leaving group, the more electron deficient the carbon, increasing the rate of reaction. The use of either the F- or Cl-monomer for PIM-1 polymerization can result in variations in the topological balance of the polymer (noted in Table 2) due to the difference in reaction rate. All polymer samples were analyzed in dilute solution using multidetector GPC. The weight average molar mass (*M*_w_), number average molar mass (*M*_n_), and dispersity (*Đ*) were determined for the soluble fraction of each polymer, as shown in Table 2. This does not represent the entire sample as colloidal network is filtered from solution prior to analysis.

**Table 2:** Experimental Conditions, Percentage Yields, *M*_w_, *M*_n_, *Đ* from GPC, Degree of Branching from ^1^H NMR and Network Content of PIM-1 Synthesized from a Conventional Solvent System and from MDDOP

polymer	reaction conditions	yield (%)	*M*_w_ (g mol^–1^)	*M*_n_ (g mol^–1^)	*Đ*	degree of branching (%)	network content (%)
conventionally synthesized PIM-1^6^	TFTPN, 160 °C, 30 min	97.2	116,000	59,500	1.9	3.7	<2
F1	TFTPN, 140 °C, 50 min	84.6	17,000	10,800	1.6	9.8	38.5
F2	TFTPN, 140 °C, 6 h	89.6	20,200	11,500	1.8	8.7	16.7
F3	TFTPN, 160 °C, 2 h	90.0	24,500	8,300	2.9	5.0	36.1
C1^a^	TCTPN, 120 °C, 6 h	n/a	n/a	n/a	n/a	n/a	n/a
C2	TCTPN, 140 °C, 6 h	90.4	70,700	37,300	1.9	14.5	11.5
C3	TCTPN, 160 °C, 2 h	90.0	44,900	25,900	1.7	6.3	18.5

a Product that could not be isolated from waste streams.

Reactions carried out using TFTPN (polymers F1–3) produced soluble polymer fractions with lower *M*_w_ of ∼20,000 g mol^–1^ compared to those synthesized using TCTPN (polymers C2–3). For polymers F1–3, polymerizations appear to proceed at a faster rate, with branching leading to significant colloidal network formation in short reaction times. It should be noted that the longer reaction time (6 h) for reaction F2 resulted in a polymer with a lower network content. PIM-1 polymerizations are reversible and depolymerization may occur from a long reaction duration. These reactions, except for reaction F1, were run for longer times at high temperatures than previously reported for PIM-1.

Soluble PIM-1 produced from the Cl-monomer (polymers C2–3) had higher *M*_w_ than polymers F1–3. Polymer C2, synthesized at 140 °C, had the highest *M*_w_ (70,700 g mol^–1^), while a decrease in *M*_w_ for Polymer C3 (44,900 g mol^–1^) was observed at a higher reaction temperature of 160 °C. The decreased network content could be a result of the slower reaction rate with TCTPN.

The multidetector GPC contained a viscosity detector, which allowed Mark–Houwink (MH) plots to be constructed. The MH plot is a double logarithmic plot of intrinsic viscosity, [η], against molar mass, *M*, developed from eq 9.
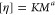
9where *K* and *a* are the MH constants.

**Figure 4:**
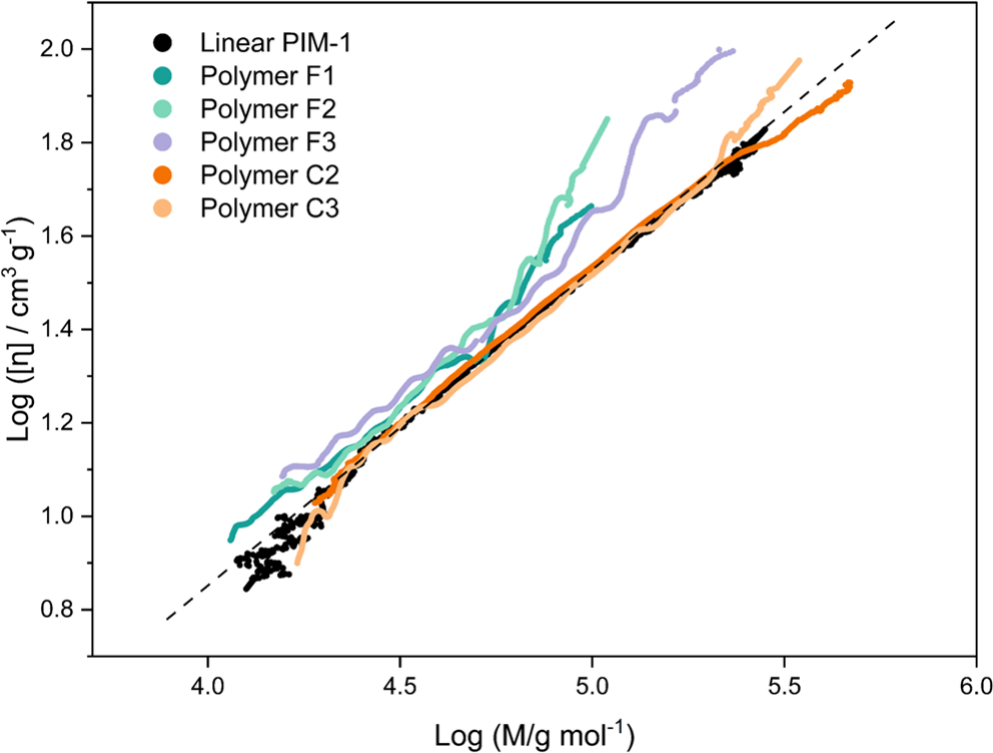
Mark–Houwink plots obtained for polymers F1–3, C2–3 in chloroform compared against a linear PIM-1 sample (black line).^7^

Figure 4 shows the MH plots of polymers compared to a disubstituted linear PIM-1.^7^ In MH plots, *a* can be determined from the gradient. A conventional linear polymer that forms a random coil exhibits an *a* value of 0.5–0.8, whereas conventional branched polymers typically exhibit lower values of *a*.^7^ When comparing conventional polymers with similar *M*, a branched polymer will have a smaller hydrodynamic volume, and hence lower intrinsic viscosity, than a linear polymer because it has a higher molecular density.^21^ However, PIMs do not show this trend, possibly because the branching can lead to an increase rather than a decrease in hydrodynamic volume. The rigidity of the polymer backbone may create a more expanded, open structure. In contrast, simple cyclic structures can exhibit a decreased intrinsic viscosity.^7^Table 3 shows the calculated *a* values for polymers F1–3 and C2–3.

**Table 3:** Calculated *a* Values for Polymers 1–5 from MH Plot

polymer	*M* range (g mol^–1^)	*a*
linear PIM-1	12,500–283,500	0.69
F1	11,500–54,800	0.63
	54,800–99,400	1.09
F2	14,900–62,300	0.69
	62,300–94,800	1.51
F3	15,600–83,800	0.66
	83,800–232,400	1.1
C2	18,900–464,000	0.64
C3	17,100–345,800	0.73

Figure 4 shows that soluble polymer fractions derived from TFTPN and TCTPN behave differently in solution. Polymers C2 and C3 (derived from TCTPN) both resemble linear PIM-1 over most of their molar mass ranges, although both have significant amounts of branching, indicated in Table 2.

Polymers F1–3 (derived from TFTPN) are found above the linear PIM-1 on the MH plot, which suggests these samples may exist in more expanded structures, causing a higher hydrodynamic volume. At higher molar mass, they show an upturn in the plot, with a higher *a* value. This suggests that structures with a high molar mass are rigid and anisotropic, as well as a potential precursor to the colloidal network material filtered from these samples.

#### 3.4.2 Branching, Network Content and BET Area

**Figure 5:**
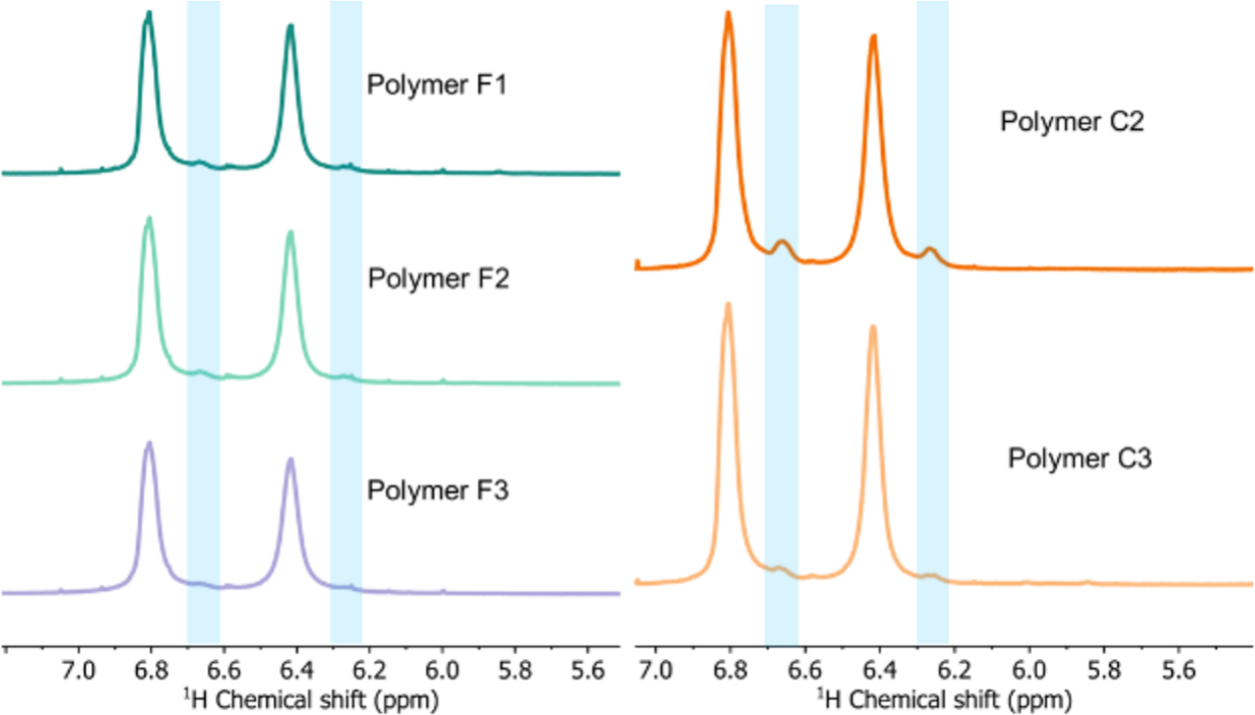
Aromatic proton regions of ^1^H NMR spectra obtained of polymers F1–3 and C2–3 in deuterated chloroform, highlighting small peaks attributable to branched structures.

Each polymer sample was analyzed via ^1^H NMR and network content analysis. Figure 5 shows the aromatic proton regions of the spectra for polymers F1–3 and C2–3. The small peaks highlighted at δ 6.66 and 6.27 ppm, are indicative of branched structures within the polymer.

Lorentzian peak fitting of these small peaks relative to the main aromatic peaks associated with disubstituted residues allows determination of the degree of branching.^19^^1^H NMR spectra of all samples are provided in Supporting Information. The levels of branching found across all the polymer samples are higher than observed in other solvent systems in polymerizations carried out at this small scale and temperature range.^7,8^ TCTPN derived polymer C2 showed both highest *M*_w_ and the highest degree of branching (14.5%), but showed less progression into colloidal network than TFTPN derived polymers. Polymer C3, prepared at a higher polymerization temperature, showed less branching than polymer C2 and network content remained lower than the equivalent TFTPN derived polymer F3.

If the filtered network material were considered in the overall molar mass distribution of the TFTPN derived polymers, the effective molar mass would be considerably higher. Other work has shown that TCTPN polymerizations proceed at a slower rate, so that loop and ring formation can occur during the step growth reactions consuming reactive ends.^8^ If this is occurring here, this would lower the progression of branched polymer into colloidal network.

Nitrogen adsorption/desorption analysis was carried out for polymers F3 and C2, giving BET areas of 666 and 762 m^2^ g^–1^, respectively. These values are within the range obtained for samples of PIM-1 synthesized in conventional solvents.

### 3.5 Pure Gas Permeation Testing

**Figure 6:**
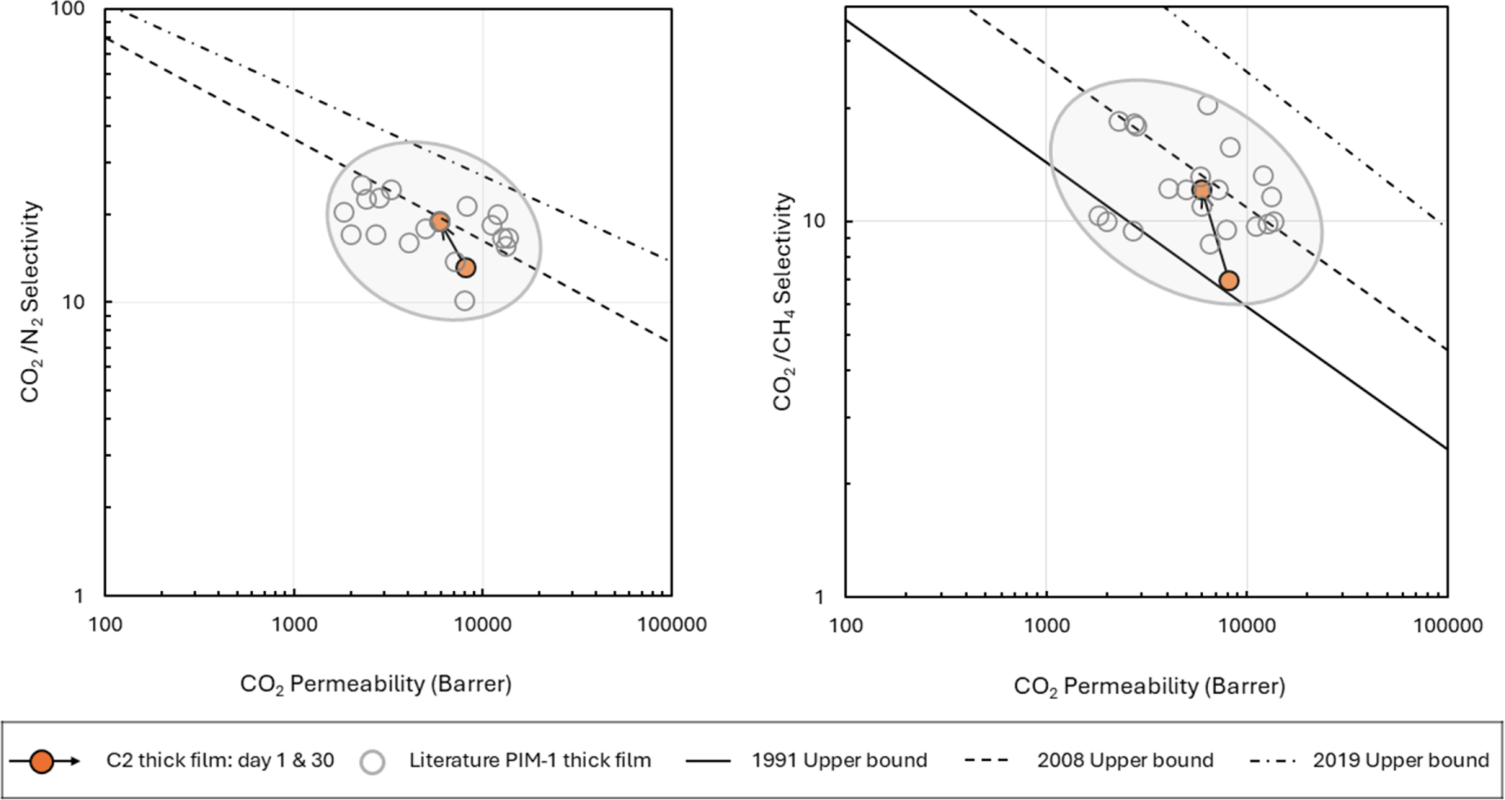
Robeson plots of ideal CO_2_/N_2_ (left plot) and CO_2_/CH_4_ (right plot) selectivities against CO_2_ permeability of polymer C2 day 1 and 30 aging data, compared with the data reported for thick PIM-1 films in the literature.^6,10,16,22−38^

Polymer C2 was fabricated into self-standing membranes of ∼45 μm thickness. Pure gas permeation testing was performed using three gases (N_2_, CH_4_, and CO_2_). The membranes were tested on days 1, 30, and 100 after preparation. In Figure 6 the 1 and 30-day aged data are shown on Robeson plots for CO_2_/N_2_ and CO_2_/CH_4_, and compared with data from the literature for self-standing membranes of PIM-1. A Robeson plot is a double logarithmic plot of selectivity against the permeability of the fastest gas of a gas pair, and it indicates the upper bound of performance based on data available for polymeric membranes at a particular point in time. It can be seen in Figure 6 that published data for PIM-1 show a high degree of scatter, but membranes cast from PIM-1 fabricated using MDDOP are within the expected range of performance for PIM-1 samples produced from conventional solvent systems. Polymer C2 exhibited a CO_2_ permeability of 8135 Barrer at day one, decreasing to 5961 Barrer after 30 days. CO_2_/N_2_ and CO_2_/CH_4_ selectivities saw increases from 13.2 to 18.8 and 6.9 to 12.1, respectively.

## 4 Conclusions

MDDOP was successfully synthesized and isolated for mass-based green metrics analysis. The reaction between methyl isobutyrate and DMAA (100% AE) does not require additional workup steps to form the desired chemical structure of the solvent. cEF and CI_total_ were low compared to those for the synthetic routes of PolarClean.^15^ Since similar chemistry is already employed in commercial synthesis of PolarClean, it is envisaged that the synthesis of MDDOP could readily be scaled-up. MDDOP was successfully used as a polar aprotic solvent in a nucleophilic aromatic substitution reaction, producing PIM-1. MDDOP may be classified as a green solvent from mass-based green metrics analysis; however, its environmental impact was not determined. If MDDOP has similar toxicology and environmental impact as those of PolarClean, it may be a viable green solvent alternative to polar aprotic solvents. PIM-1 suitable for membrane formation was synthesized via polymerization with TCTPN in MDDOP at 140 °C for 6 h. In MDDOP, TCTPN provided soluble PIM-1 with a higher soluble molar mass polymer and lower network content than TFTPN. Polymeric samples with higher *M*_w_ could be produced by further optimization of the reaction conditions, including shorter reaction times with TCTPN. Using MDDOP as an alternative solvent, the environmental impact can be reduced, as it can be easily recovered from waste streams. The solvent recovery of methanol and MDDOP reduced the overall solvent waste produced by 22%. A polymer synthesized in MDDOP was fabricated into self-standing membranes and tested with pure gases for CO_2_/N_2_ and CO_2_/CH_4_ separations, performing similarly to PIM-1 obtained from conventional solvents. This work points the way toward greener synthesis of PIMs, enhancing the overall sustainability of PIM-based membranes for energy-efficient separations.

## Data Availability

Data supporting this study are available within the Article and the Supporting Information.

## Supporting Information Available

Green metrics analysis data, TTSBI purification, solvent isolation parameters, GC-MS, Mark–Houwink parameters, NMR, DLS, elemental analysis, MALDI-TOF, N_2_ adsorption and desorption, and single gas testing data (PDF)

## Abbreviations

PIM: polymer of intrinsic microporosity
MDDOP: methyl-5-(dimethylamino)-2,2-dimethyl-5-oxopentanoate
TTSBI: 5,5′,6,6′-tetrahydroxy-3,3,3′,3′-tetramethyl-1,1′-spirobisindane
TFTPN: tetrafluoroterephthalonitrile
TCTPN: tetrachloroterephthalonitrile
SDGs: sustainable development goals
IM: intrinsic microporosity
AE: atom economy
cEF: complete environmental factor
CI_t__otal_: total carbon intensity
DMAc: dimethylacetamide
IMI: Innovative Medicines Initiative
GPC: gel permeation chromatography
NMR: nuclear magnetic resonance
MALDI-TOF: matrix-assisted laser desorption ionization-time-of-flight
DLS: dynamic light scattering
