# Supplementary material for: Greener Synthesis of the Polymer of Intrinsic Microporosity PIM-1 for Gas Separation
Source: ACS Sustain Chem Eng. 2024 Dec 31;13(7):2784–92. doi: 10.1021/acssuschemeng.4c08475 (PMC11863552; doi:10.1021/acssuschemeng.4c08475)
Supplement: Supplementary file 1 — sc4c08475_si_001.pdf [file sc4c08475_si_001.pdf]

Greener Synthesis of the Polymer of Intrinsic Microporosity PIM-1 for Gas Separation

Supporting Information

Alisha Ayyaz[a]*, Andrew B. Foster[a], Levente Cseri[b], Gyorgy Szekely[b,c], Peter M. Budd[a]*.

Pages: 20

Figures: 21

Tables: 9

[a.] *Department of Chemistry, University of Manchester, Manchester, M13 9PL, UK.*
[b.] *Department of Chemical Engineering, University of Manchester, Manchester, M13 9PL, UK.*
[c.] *King Abdullah University of Science and Technology (KAUST), Kingdom of Saudi Arabia*

Table of Contents

1. Greener Synthesis of PIM-1: Mass Based Green Metrics Analysis Data....4

Table S1: Routes for the formation of PolarClean[1] 4

Table S2: Reagents required, and chemicals produced for the synthesis of 1 kg of MDDOP, theoretical carbon intensity (CI) from complete combustion. 4

Table S3: Process steps contributing to carbon intensity of energy consumption (CI_{ec}) and carbon intensity of cooling water (CI_{wc}) of 100 g scale synthesis of MDDOP. 4

Figure S1: Cumulative atom economy percentage, complete environmental factor (cEF), and total carbon intensity (CI_{total}) of patented and improved routes to PolarClean[15] and MDDOP. 5

2. TTSBI Purification 5

3. Solvent isolation – reduced pressure distillation 5

Figure S2: Schematic of reduced pressure distillation equipment. 6

4. Gas Chromatography 6

Figure S3: GC-MS chromatogram of MDDOP in a 1 mg ml^{-1} solution in dichloromethane, with a table of components in insert. 6

5. Mass Spectrometry 7

Figure S4: Mass spectrum of MDDOP. 7

NMR of recovered methanol....7

Figure S5: Proton NMR of pure and recovered methanol in $CDCl_3$. 7

6. GPC Mark-Houwink parameters 8

Table S4: Calculated *a* and *K* values for polymers F1-3 and C2-3 from MH plot. 8

7. ^{1}H NMR of PIM-1 polymers F1-3 and C2-3 8

Table S5: ^{1}H NMR expected structures and peaks for PIM-1 polymers in deuterated chloroform 8

Figure S6: ^{1}H NMR of polymer F1. 9

Figure S7: ^{1}H NMR of polymer F2. 9

Figure S8: ^{1}H NMR of polymer F3. 10

Figure S9: ^{1}H NMR of polymer C2. 10

Figure S10: ^{1}H NMR of polymer C3. 11

8. Lorentzian fitting and branching quantification 12

Figure S11: Lorentzian fitting of polymers F1-3 and C2-3 in aromatic proton region on ^{1}H NMR. 12

Table S6: Lorentzian fitting values and branching quantification for polymers F1-3 and C2-3 13

9. DLS number and intensity distribution of hydrodynamic diameter for polymers F1-3 and C2-3. 13

Figure S12: Number distributions of hydrodynamic diameter determined via DLS for polymers F1-3 and C2-3 in chloroform. 13

Figure S13: Intensity distributions of hydrodynamic diameter from DLS for 50 ppm solutions in chloroform of PIM-1 polymers F1-3 and C2-3. 14

10. Elemental Analysis 14

Table S7: Elemental Analysis of the PIM-1 polymers F1-3 and C2-3 14

11. MALDI-TOF spectra 14

Figure S14: MALDI-TOF spectrum of PIM-1 sample polymer F1 15

Figure S15: MALDI-TOF spectrum of PIM-1 sample polymer F2 15

Figure S16: MALDI-TOF spectrum of PIM-1 sample polymer F3 16

Figure S17: MALDI-TOF spectrum of PIM-1 sample polymer C2 16

Figure S18: MALDI-TOF spectrum of PIM-1 sample polymer C3 17

12. BET Surface area analysis 17

Figure 19: N_2 adsorption and desorption isotherms of polymers F3 and C2, and surface area from BET analysis. 17

13. Single Gas Testing Data 18

Figure S20: Schematic of membrane coupon cutting and single gas testing apparatus 18

Table S8: Gas permeation data for N_2, CH_4 and CO_2 of polymer C2 18

Table S9: Gas permeation data for N_2, CH_4 and CO_2 for PIM-1 from literature, shown in Figure 6. 18

Permeance of a coupon 19

Calculating gas pair selectivity 20

14. References 20

1. Greener Synthesis of PIM-1: Mass Based Green Metrics Analysis Data

Table S1: Routes for the formation of PolarClean[1]

Route	Atom Economy (%)	Complete E-factor (%)	Total Carbon Intensity (kg kg^{-1})
		Patented routes	
A1	22.8	65.3	222
A2	23.5	31.9	169
A3	13.5	17.5	112
		'Improved' routes	
B	62.6	83.6	145
C	5.6	10.2	29

Table S2: Reagents required, and chemicals produced for the synthesis of 1 kg of MDDOP, theoretical carbon intensity (CI) from complete combustion.

Reagents	Mass / kg	Molar mass / g mol^{-1}	Molar amount / mol	Molar carbon content / mol mol^{-1}	Carbon content / mol	CI / kg kg^{-1}
Methyl isobutyrate	0.65	102.13	6.36	5	31.82	1.40
N,N-Dimethylacrylamide	0.57	99.13	5.78	5	28.92	1.27
KOtBu	0.02	111.21	0.19	4	0.77	0.03
Oxalic acid	0.03	90.03	0.33	2	0.65	0.03
H_2O	0.33	18.02	18.04	0	0.00	0.00
**Products**						
MDDOP	1	201.26	4.97	10	49.69	-2.19

Table S3: Process steps contributing to carbon intensity of energy consumption (CI_{ec}) and carbon intensity of cooling water (CI_{wc}) of 100 g scale synthesis of MDDOP.

	Duration/ h				
**Process step**	**vacuum**	**stirring**	**heating**	**chiller**	**water cooling**
Reaction	0	1	0	1	0
Vacuum distillation	3	3	3	0	3
Total time/ h	3	4	3	1	3
Total E/ kWh	0.405	0.06	3	1.8	na
Total volume/ m^3	na	na	na	na	0.45
CI/ kg kg^{-1}	1.24	0.18	9.22	5.53	1.55

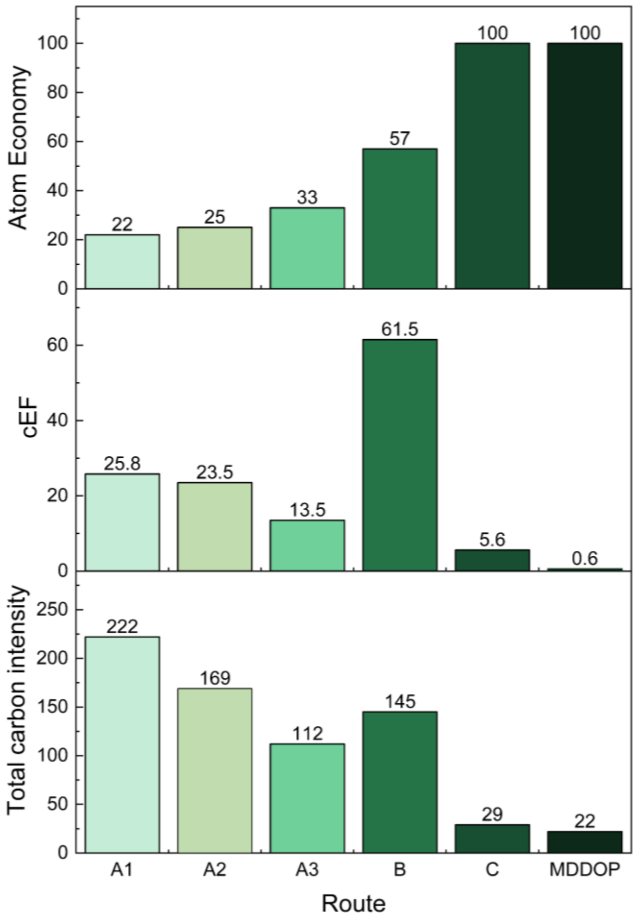

Figure S1: Cumulative atom economy percentage, complete environmental factor (cEF), and total carbon intensity (CI_{total}) of patented and improved routes to PolarClean[1] and MDDOP.

2. TTSBI Purification

To a 3 L round-bottom flask TTSBI (40 g) was added, and a 3-neck connector was fitted. A reflux condenser and nitrogen inlet were connected. Ethyl acetate (667 mL) was added, and the mixture was refluxed at 90 °C until dissolution of TTSBI neared completion (approximately 3 h). Hexane (667 mL) was added while under reflux and stirred for 10 min. The solution was cooled to room temperature using an ice-bath. The mixture was filtered using a sintered funnel and dried for 24 h in a vacuum desiccator.

3. Solvent isolation – reduced pressure distillation

MDDOP was isolated using reduced pressure distillation via the set up illustrated in figure S1. Water was collected at 21°C and MDDOP was collected at 132°C. Vacuum pressure could not be recorded using this method, calculated pressure was approximately 8 mbar (calculated using the Clausius-Clapeyron equation, assuming the heat of vaporization is constant over a pressure range). When initially synthesized the parameters stated the distillate collected at 120 °C and 3.6 mbar.[1]

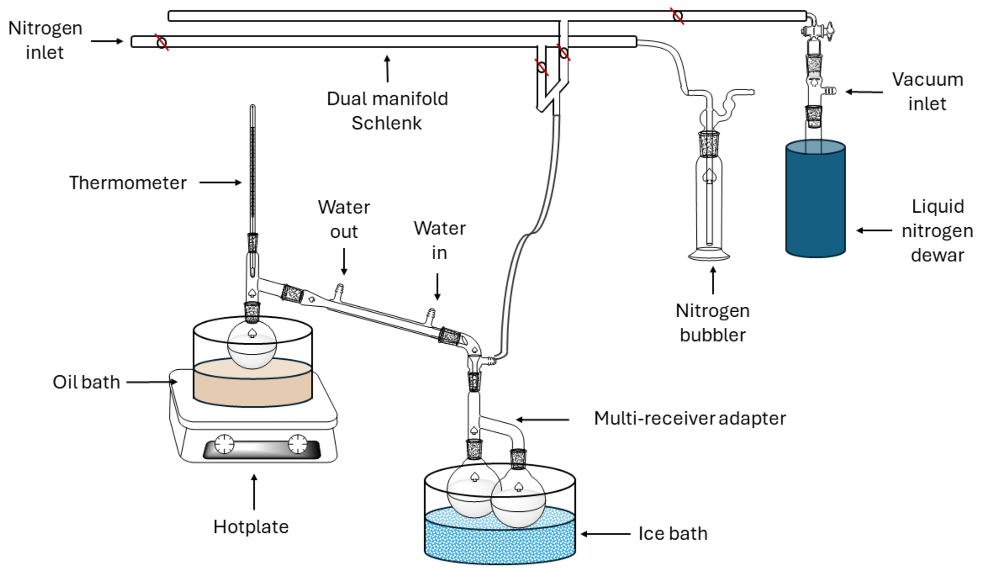

Figure S2: Schematic of reduced pressure distillation equipment.

4. Gas Chromatography

GC-MS analysis was carried out using a Shimadzu GCMS-QP210 SE. A sample of 1 µl was injected into the GC column 30 m x 0.25 mm x 25 um film thickness, with helium gas flux of 1 ml min^{-1}, injection split 20:1 at 300 °C. Heating was from 50 °C (3 min) to 300 °C (5 min) with a heating rate of 25 °C min^{-1}. MS analysis parameters were EI 70 eV, ionization scan 50–650 g mol^{-1}, scan time of 2.70–19 min and temperatures of ion source and transfer line were 230 °C and 320 °C, respectively. The recovered methanol was made into a 1 mg ml^{-1} solution in chloroform.

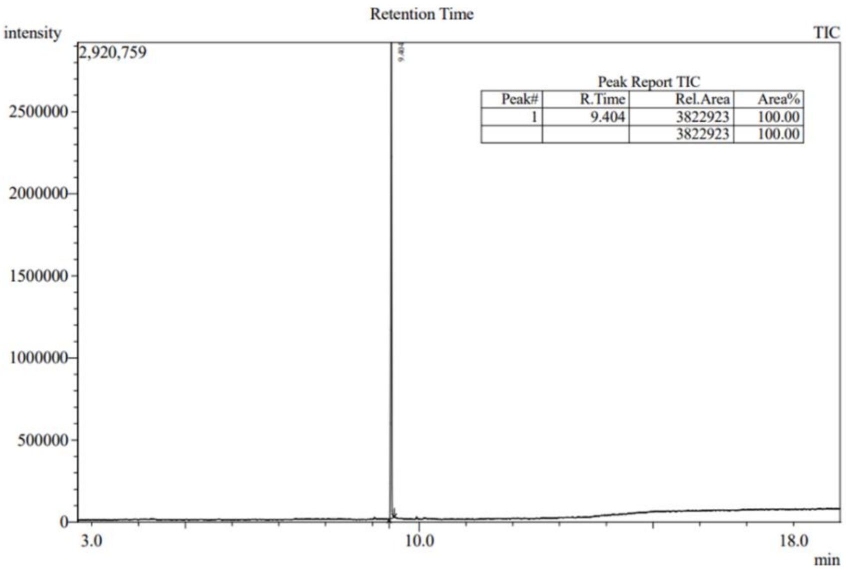

Figure S3: GC-MS chromatogram of MDDOP in a 1 mg ml^{-1} solution in dichloromethane, with a table of components in insert.

5. Mass Spectrometry

The GS-MS methodology is described in section 4.

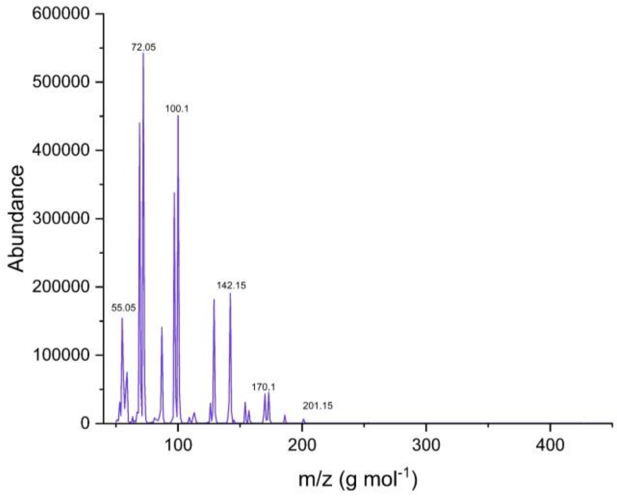

Figure S4: Mass spectrum of MDDOP.

NMR of recovered methanol

^{1}H NMR spectrum of recovered methanol was recorded using a Bruker Avance II 500 MHz instrument. Polymer samples were prepared with a concentration of 10 mg ml^{-1} in $CDCl_3$ for the NMR analysis. Signal peaks for the solvent were used as references.

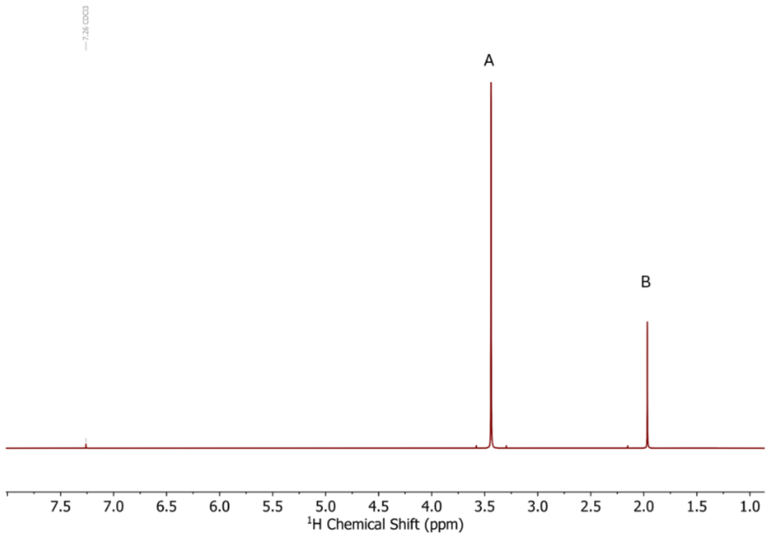

Figure S5: Proton NMR of pure and recovered methanol in $CDCl_3$.

*Recovered methanol resonances: (δ, ^{1}H NMR, $CDCl_3$): A - aliphatic C**H**, 3.44 ppm (3 protons); B – aliphatic alcohol O**H**, 1.97 ppm (1 proton); small peak at 2.15 ppm is due to the presence of residual methanol from washing glassware before use.*

6. GPC Mark-Houwink parameters

Table S4: Calculated *a* and *K* values for polymers F1-3 and C2-3 from MH plot.

Polymer	*M* range (g mol^{-1})	*a*	*K* (cm^3 g^{-1})
Linear PIM-1	12,500–283,500	0.69	1.2×10^{-2}
F1	11,500–54,800	0.63	2.4×10^{-2}
	54,800–99,400	1.09	1.8×10^{-4}
F2	14,900–62,300	0.69	1.3×10^{-2}
	62,300–94,800	1.51	1.6×10^{-6}
F3	15,600–83,800	0.66	1.9×10^{-2}
	83,800–232,400	1.1	1.4×10^{-4}
C2	18,900–464,000	0.64	2.1×10^{-2}
C3	17,100–345,800	0.73	7.9×10^{-3}

7. ^{1}H NMR of PIM-1 polymers F1-3 and C2-3

^{1}H NMR spectra of PIM-1 polymers were recorded using a Bruker Avance II 500 MHz instrument. Polymer solutions of 20 mg ml^{-1} in $CDCl_3$ were prepared for the NMR analysis. Signal peaks for the solvent were used as references.

Table S5: ^{1}H NMR expected structures and peaks for PIM-1 polymers in deuterated chloroform

Di-substituted main chain

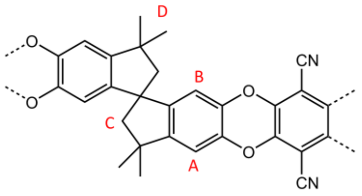

A: aromatic C**H**, s, δ 6.81 ppm (2 protons)
B: aromatic C**H**, s, δ 6.42 ppm (2 protons)
C: aliphatic C**H$_2$**, dd, δ 2.16-2.33 ppm (4 protons)
D: aliphatic C**H$_3$**, d, δ 1.31-1.37 ppm (12 protons)

**Under-reacted residue**	**Branched residue**
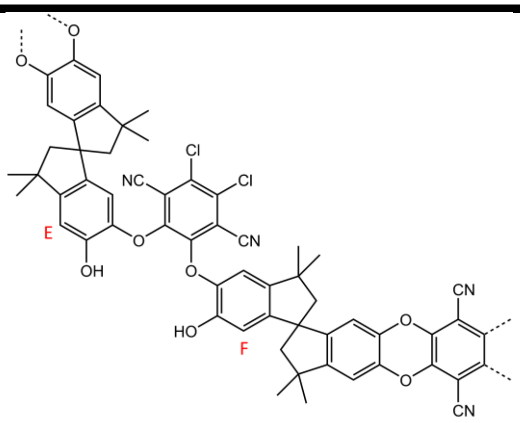	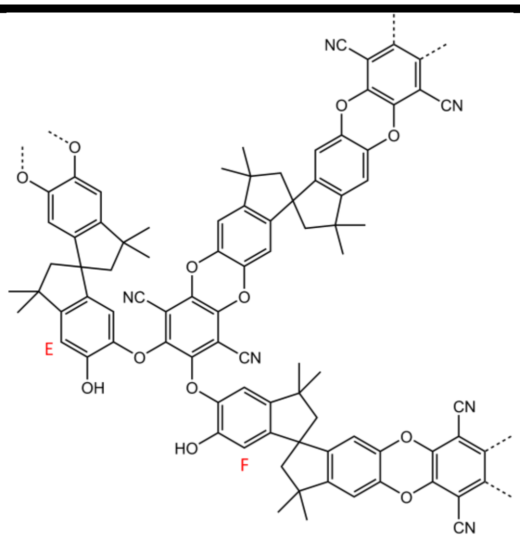

Aromatic C**H**, δ 6.66 and 6.27 ppm

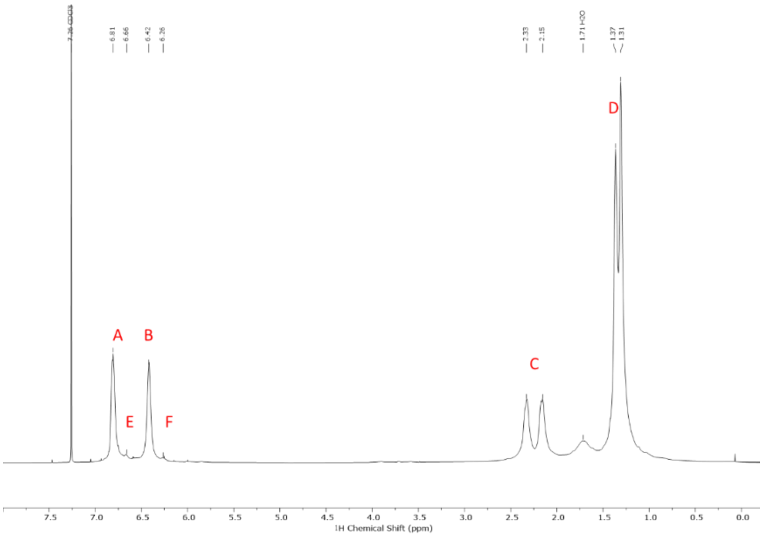

Figure S6: ^{1}H NMR of polymer F1.

PIM-1 Main chain resonances: *(δ, ^{1}H NMR, $CDCl_3$): A - aromatic C**H**, 6.81 ppm (2 protons); B - aromatic **CH,** 6.42 ppm (2 protons); C -aliphatic C**H**$_2$, 2.33 & 2.15 ppm (4 protons); D - aliphatic C**H**$_3$ × 1.37 & 1.31 ppm (12 protons).* ***PIM-1 Branching/ Underreacted chain ends:*** *(δ, ^{1}H NMR, $CDCl_3$): E - aromatic C**H**, 6.66 ppm; F – aromatic C**H**, 6.27 ppm.*

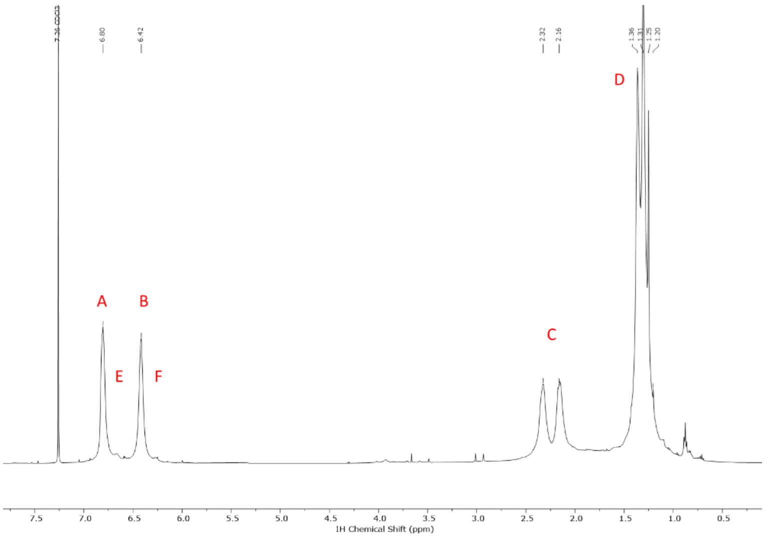

Figure S7: ^{1}H NMR of polymer F2.

PIM-1 Main chain resonances: *(δ, ^{1}H NMR, $CDCl_3$): A - aromatic C**H**, 6.80 ppm (2 protons); B - aromatic **CH,** 6.42 ppm (2 protons); C -aliphatic C**H**$_2$, 2.32 & 2.16 ppm (4 protons); D - aliphatic C**H**$_3$ × 1.36 & 1.31 ppm (12 protons).* ***PIM-1 Branching/ Underreacted chain ends:*** *(δ, ^{1}H NMR, $CDCl_3$): E - aromatic C**H**, 6.66 ppm; F – aromatic C**H**, 6.27 ppm.*

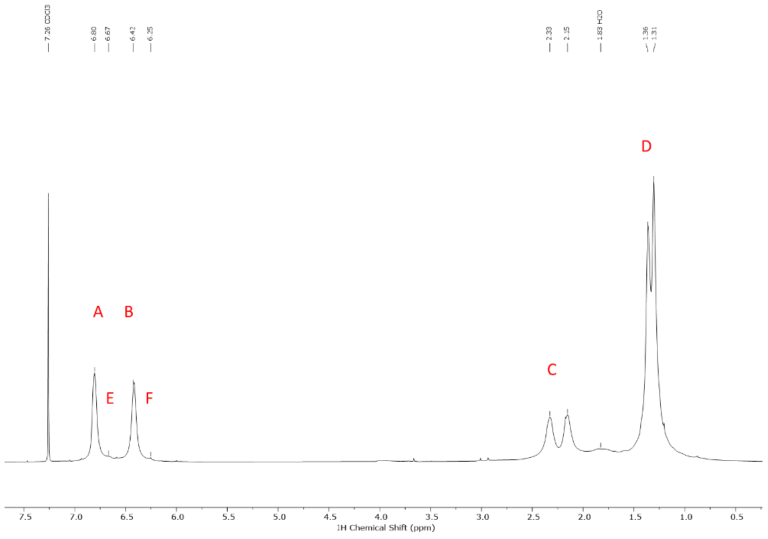

Figure S8: ^{1}H NMR of polymer F3.

PIM-1 Main chain resonances: *(δ, ^{1}H NMR, $CDCl_3$): A - aromatic C**H**, 6.80 ppm (2 protons); B - aromatic **CH,** 6.42 ppm (2 protons); C -aliphatic C**H**$_2$, 2.33 & 2.15 ppm (4 protons); D - aliphatic C**H**$_3$ × 1.36 & 1.31 ppm (12 protons).* ***PIM-1 Branching/ Underreacted chain ends:*** *(δ, ^{1}H NMR, $CDCl_3$): E - aromatic C**H**, 6.67 ppm; F – aromatic C**H**, 6.25 ppm.*

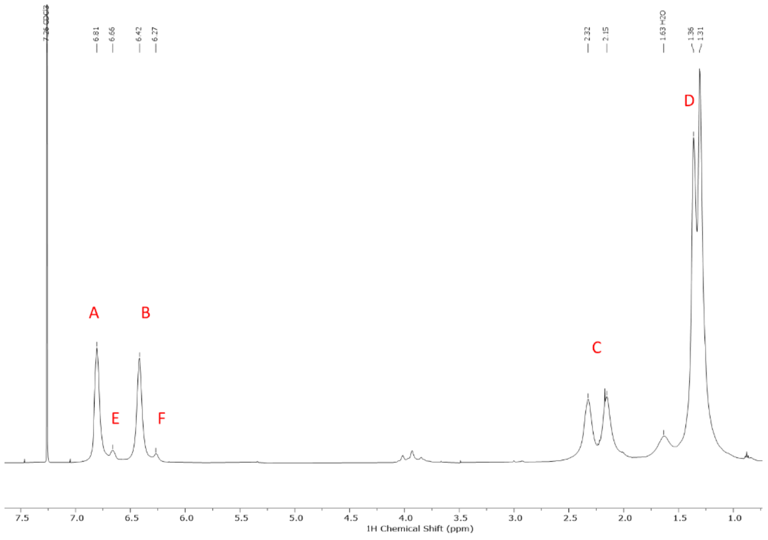

Figure S9: ^{1}H NMR of polymer C2.

PIM-1 Main chain resonances: *(δ, ^{1}H NMR, $CDCl_3$): A - aromatic C**H**, 6.81 ppm (2 protons); B - aromatic **CH,** 6.42 ppm (2 protons); C -aliphatic C**H**$_2$, 2.32 & 2.15 ppm (4 protons); D - aliphatic C**H**$_3$ × 1.36 & 1.31 ppm (12 protons).* ***PIM-1 Branching/ Underreacted chain ends:*** *(δ, ^{1}H NMR, $CDCl_3$): E - aromatic C**H**, 6.66 ppm; F – aromatic C**H**, 6.27 ppm.*

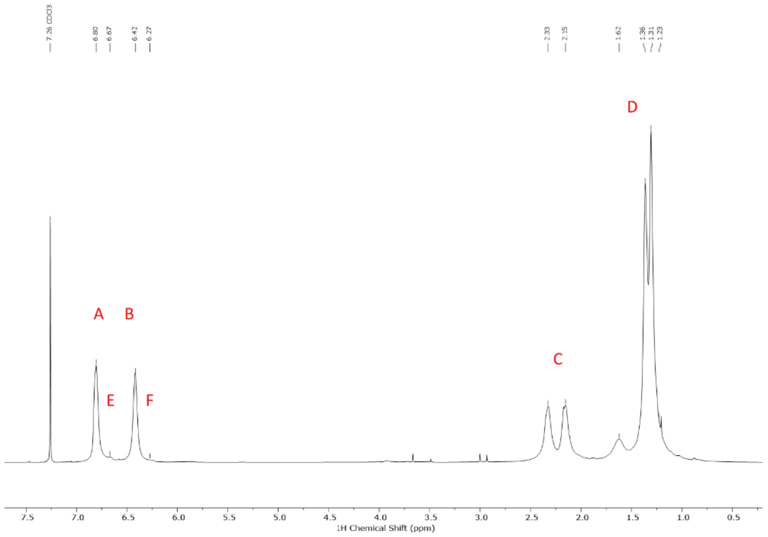

Figure S10: ^{1}H NMR of polymer C3.

PIM-1 Main chain resonances: *(δ, ^{1}H NMR, $CDCl_3$): A - aromatic C**H**, 6.80 ppm (2 protons); B - aromatic* ***CH,*** *6.42 ppm (2 protons); C -aliphatic C**H**$_2$, 2.33 & 2.15 ppm (4 protons); D - aliphatic C**H**$_3$ × 1.36 & 1.31 ppm (12 protons).* ***PIM-1 Branching/ Underreacted chain ends:*** *(δ, ^{1}H NMR, $CDCl_3$): E - aromatic C**H**, 6.67 ppm; F – aromatic C**H**, 6.27 ppm.*

8. Lorentzian fitting and branching quantification

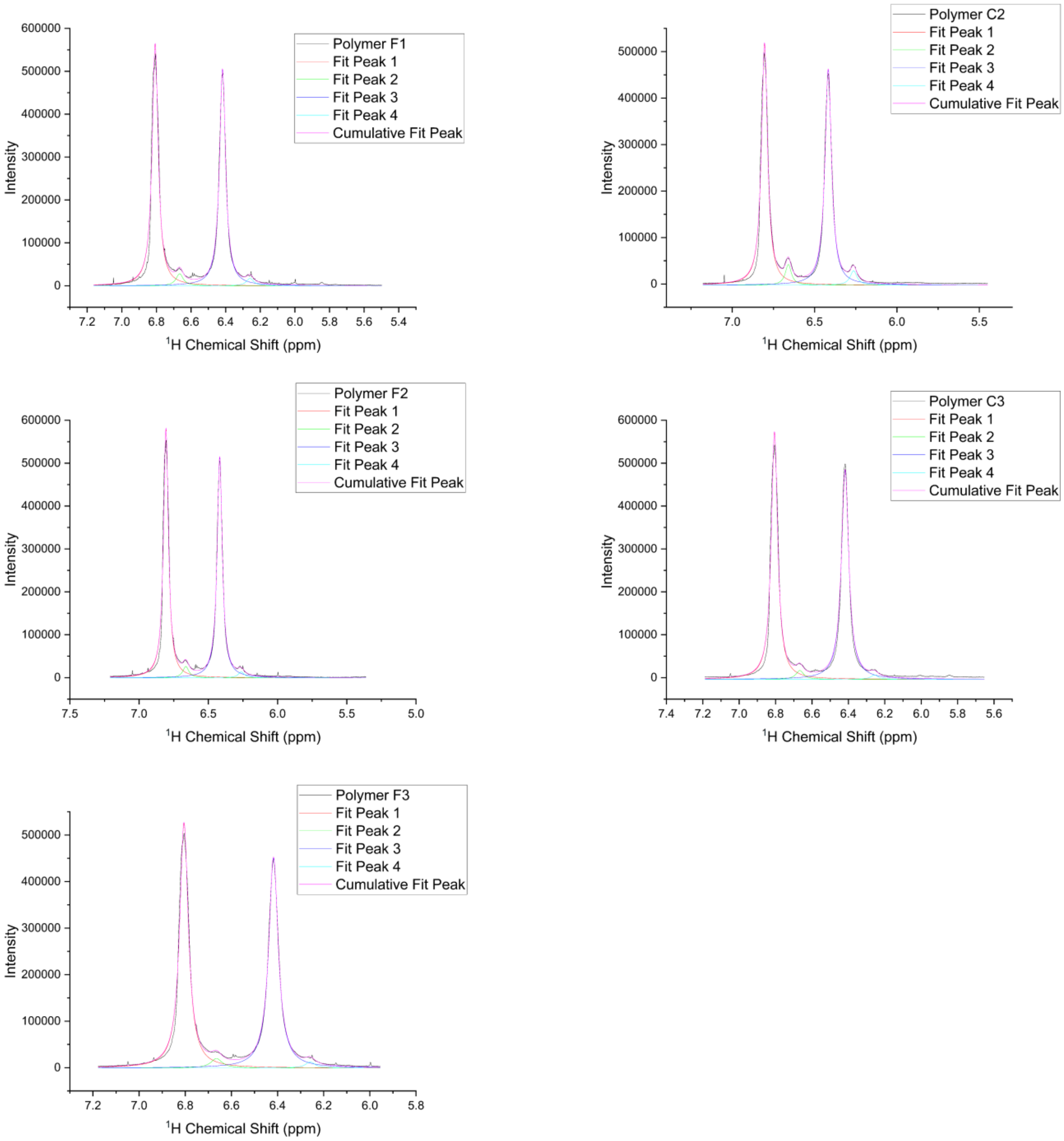

Figure S11: Lorentzian fitting of polymers F1-3 and C2-3 in aromatic proton region on ^{1}H NMR.

Table S6: Lorentzian fitting values and branching quantification for polymers F1-3 and C2-3

Polymer	Peak	Area	I_t PIM-1	I_t Branch	Branch %	PIM-1 residues per branch point
Polymer F1	1	36577	66001.342	7157.075	9.8	9
	2	2204				
	3	33003				
	4	1375				
Polymer F2	1	38624	70725.099	6709.570	8.7	11
	2	2196				
	3	35455				
	4	1159				
Polymer F3	1	37363	73690.278	3907.489	5.0	19
	2	1493				
	3	38281				
	4	461				
Polymer C2	1	35948	64891.341	11037.341	14.5	6
	2	3097				
	3	34462				
	4	2421				
Polymer C3	1	37927	72220.032	4880.113	6.3	15
	2	1661				
	3	36733				
	4	779				

9. DLS number and intensity distribution of hydrodynamic diameter for polymers F1-3 and C2-3

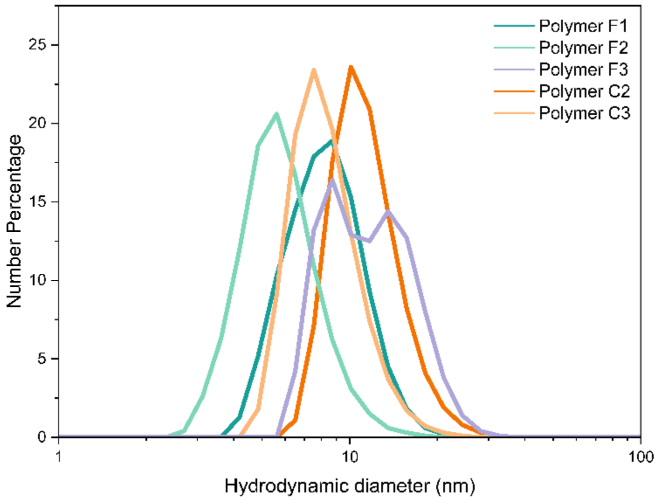

Figure S12: Number distributions of hydrodynamic diameter determined via DLS for polymers F1-3 and C2-3 in chloroform.

DLS analysis indicated that each polymer was predominantly made up of structures with a number-average hydrodynamic diameter of 5–10 nm, with polymers F1-2 and C2-3 showing a single peak around this region shown in figure 12. Polymer F3 showed a different bimodal distribution with a significant proportion of larger size structures. This polymer also had the largest polydispersity from GPC, suggesting polymer F3 has a more varied topology than the other samples.

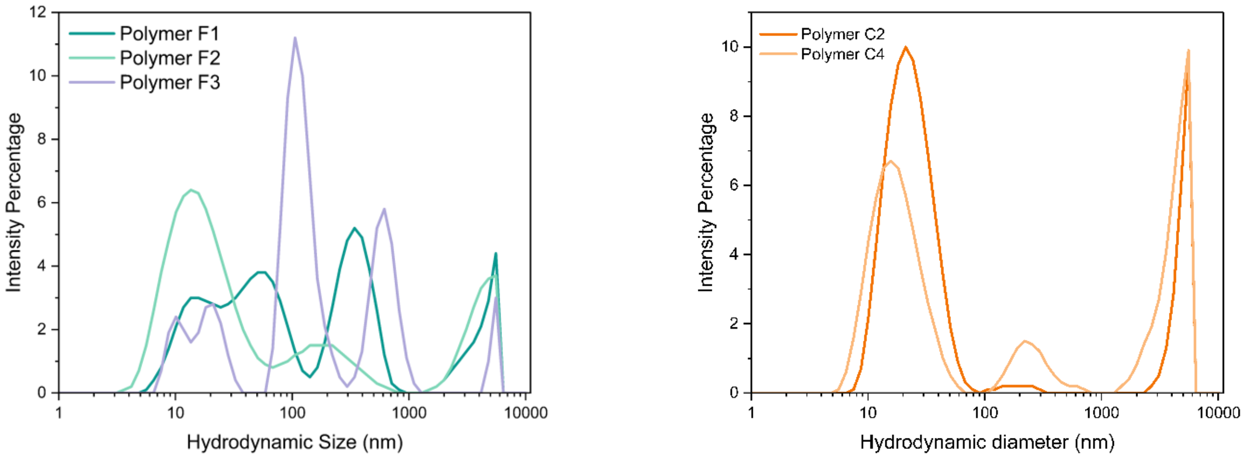

Figure S13: Intensity distributions of hydrodynamic diameter from DLS for 50 ppm solutions in chloroform of PIM-1 polymers F1-3 and C2-3.

10. Elemental Analysis

Table S7: Elemental Analysis of the PIM-1 polymers F1-3 and C2-3

Polymer sample	Reaction conditions	C (%)	N (%)	H (%)	Cl (%)	C/N	C/H	N/H	*r*(T/S)
	Cyclic PIM-1 Composition[2]	75.64	6.08	4.38	0	12.44	17.27	1.39	1.00
**F1**	F-monomer, *T*=140 °C, 50 minutes	72.44	5.71	4.36	*NA*	12.69	16.61	1.31	0.98
**F2**	F-monomer, *T*=140 °C, 6 hours	70.10	5.70	4.06	*NA*	12.30	17.27	1.40	1.01
**F3**	F-monomer, *T*=160 °C, 2 hours	72.69	6.05	4.23	*NA*	12.01	17.18	1.43	1.04
**C2**	Cl-monomer, *T*=140 °C, 6 hours	73.62	5.97	4.31	0	12.33	17.08	1.39	1.01
**C3**	Cl-monomer, *T*=160 °C, 2 hours	69.33	5.57	3.39	0	12.45	20.45	1.64	0.99

11. MALDI-TOF spectra

MALDI-TOF mass spectrometry analysis was carried out using a Shimadzu Biotech Axima instrument. Each PIM-1 polymer sample (5 mg) was dissolved in chloroform (100 µL) and mixed with a matrix solution (dithranol in THF, 10 mg ml^{-1}) in a 1:10 ratio (sample : matrix). This mixture was spotted onto a plate with sodium iodide solution (10 mg mL^{-1}) applying the layered method. Calibration was carried out with the use of a spherical peptide mix (range 1600-3500 Da). Samples were run using linear mode with pulse extraction optimised as 7000 kg mol^{-1}.

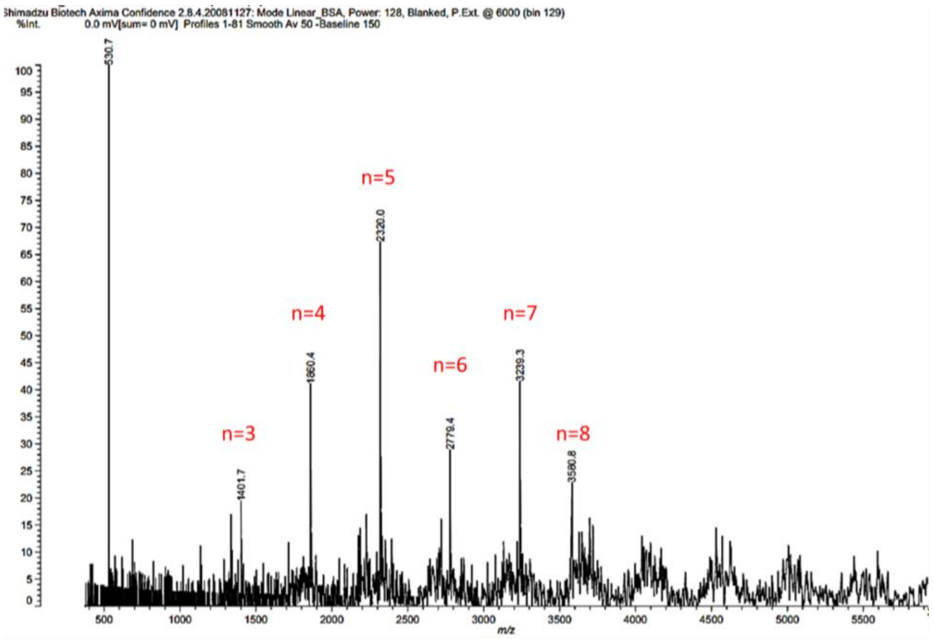

Figure S14: MALDI-TOF spectrum of PIM-1 sample polymer F1

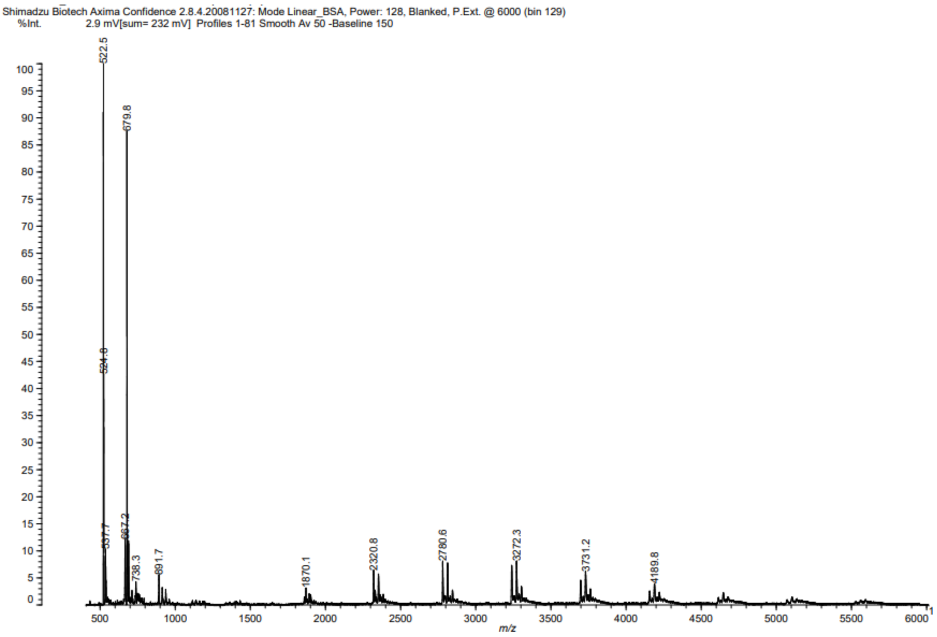

Figure S15: MALDI-TOF spectrum of PIM-1 sample polymer F2

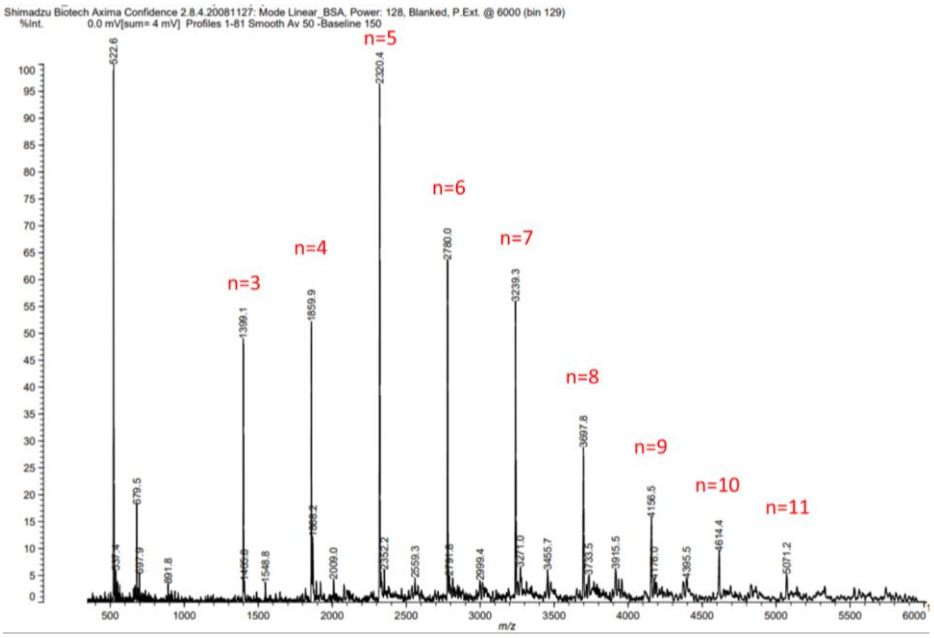

Figure S16: MALDI-TOF spectrum of PIM-1 sample polymer F3

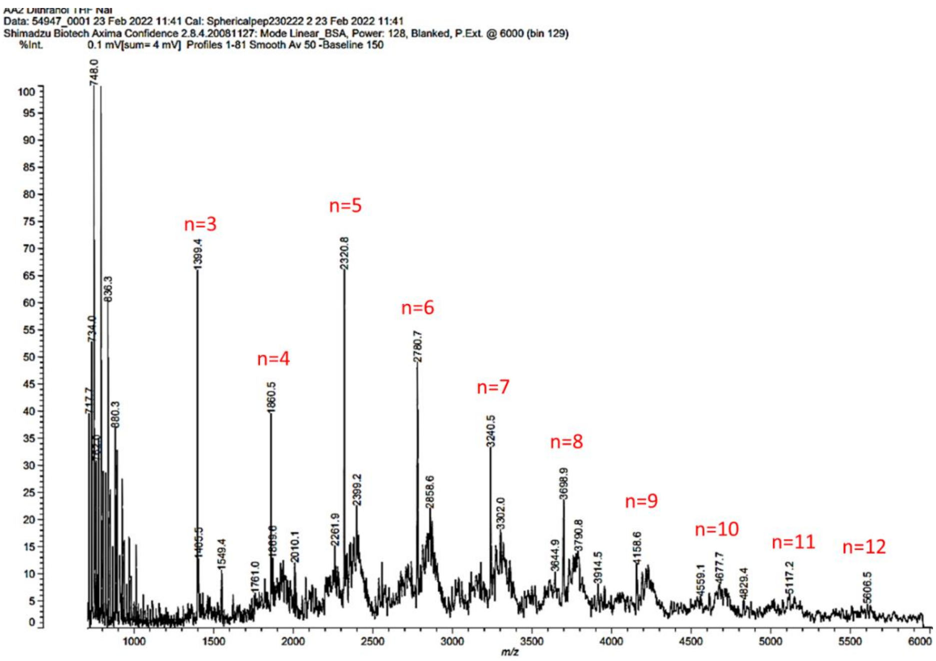

Figure S17: MALDI-TOF spectrum of PIM-1 sample polymer C2

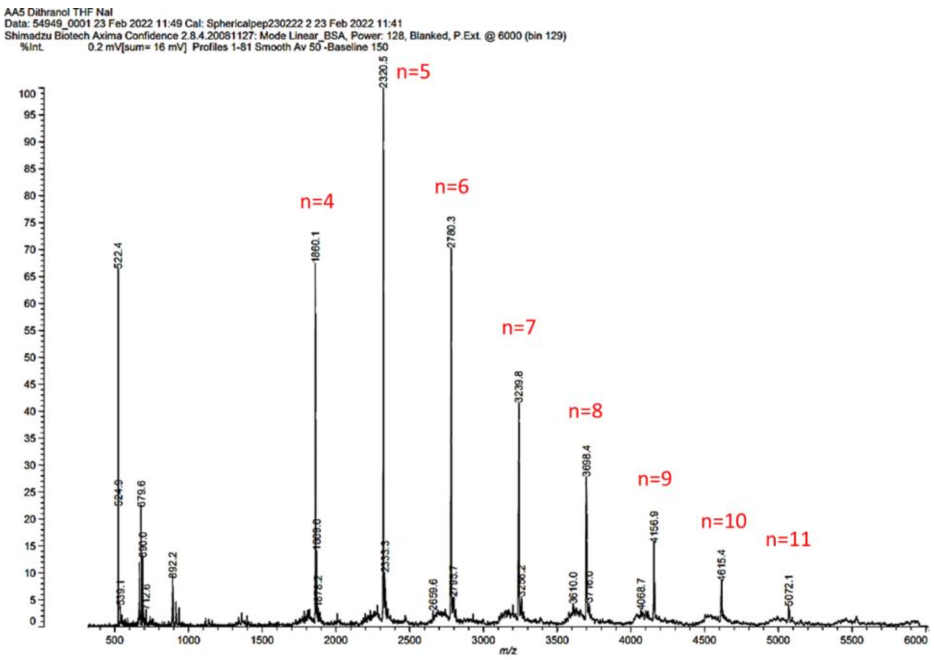

Figure S18: MALDI-TOF spectrum of PIM-1 sample polymer C3

12. BET Surface area analysis

Nitrogen (N_2) adsorption and desorption isotherms of materials were measured using a Micromeritics ASAP 2020 physisorption analyser. The sample (about 100 mg) was degassed at 120°C under a vacuum (0.1 mmHg) for 16 h before the physisorption analysis at the liquid nitrogen temperature of −196 °C (77 K). The apparent specific surface area of the materials was determined using the Brunauer–Emmett–Teller (BET) method.

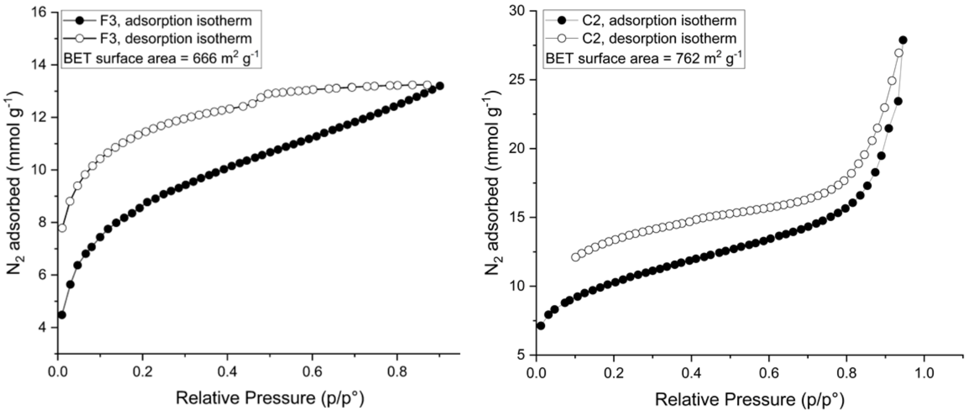

Figure 19: N_2 adsorption and desorption isotherms of polymers F3 and C2, and surface area from BET analysis.

13. Single Gas Testing Data

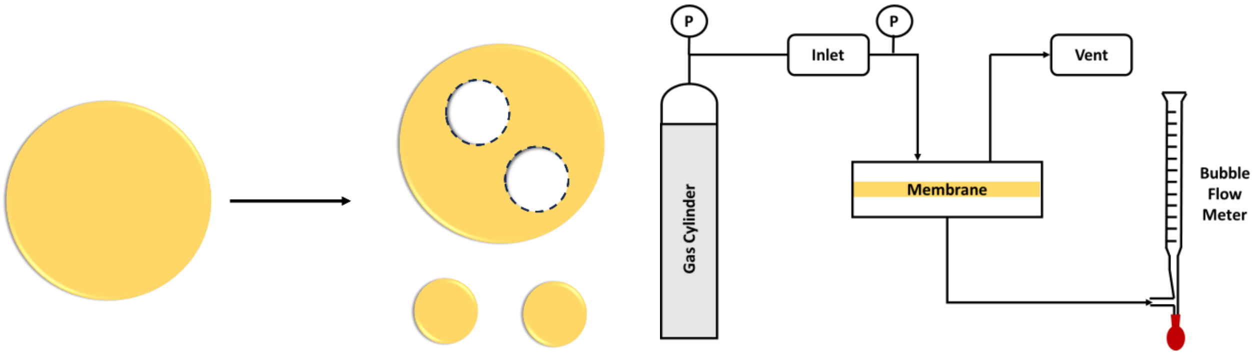

Figure S20: Schematic of membrane coupon cutting and single gas testing apparatus

Circular coupons (diameter = 19 mm) were cut from the thick films, measuring the thickness of each before gas testing using N_2, CH_4 and CO_2 consecutively. Once the membrane was placed, before each set of gas measurements was carried out, the gas pressure was set to 35 psi (N_2 and CH_4) or 25 psi (CO_2) and left to purge for 15-20 minutes. Testing was carried out on day 1, 30, and 100. Coupons were retested if necessary. The coupons have an effective area of 2.84 cm^2, they are placed into the permeation cell with a rubber ring seal. The time taken for a bubble of gas to move a given distance was recorded for each gas. At least six measurements were taken, per coupon, per gas.

Table S8: Gas permeation data for N_2, CH_4 and CO_2 of polymer C2

Coupon	Thickness (µm)	Film Aging (Days)	Permeance, *K* / GPU			Selectivity	
			N_2	CH_4	CO_2	CO_2/N_2	CO_2/CH_4
1	77.1	1	2.37 (± 0.12)	4.82 (± 0.15)	36.48 (± 2.84)	15.4	7.6
		30	3.06 (± 0.10)	5.19 (± 0.15)	49.99 (± 0.79)	16.3	9.6
		100	2.98 (± 0.00)	3.38 (± 0.15)	61.77 (± 4.19)	20.8	18.3
2	77.1	1	3.68 (± 0.08)	7.07 (± 0.14)	50.53 (± 3.05)	13.7	7.1
		30	*n/a*	*n/a*	*n/a*	*n/a*	*n/a*
		100	*n/a*	*n/a*	*n/a*	*n/a*	*n/a*
3	44.7	1	12.56 (± 0.15)	24.46 (± 0.32)	174.00 (± 8.67)	13.9	7.1
		30	7.27 (± 0.44)	10.50 (± 0.66)	119.15 (± 3.94)	16.4	11.3
		100	*n/a*	*n/a*	*n/a*	*n/a*	*n/a*
4	44.7	1	15.21 (± 0.43)	28.10 (± 1.03)	189.99 (± 6.11)	12.5	6.8
		30	6.93 (± 0.84)	11.51 (± 0.27)	147.58 (± 5.72)	21.3	12.8
		100	*n/a*	*n/a*	*n/a*	*n/a*	*n/a*

Table S9: Gas permeation data for N_2, CH_4 and CO_2 for PIM-1 from literature, shown in Figure 6.

Reference as seen in figure 6	CO_2 permeance (GPU)	CO_2/N_2	CO_2/CH_4
10	2000	17	10
	2700	17	9.4

25	1830	20.3	10.3
28	11200	18.4	9.7
29	12100	19.9	13.2
30	13600	16.5	10
31	7,183	13.7	12.1
	7,981	10.1	9.5
32	6,578	-	8.7
33	2,755	-	18.1
34	2437	22.4	-
35	3292	24.2	-
36	5920	-	13.1
37	5940	18.9	10.9
	12800	16.6	9.8
38	6400	-	20.3
39	4970	17.8	12.1
	13,300	15.5	11.6
	2840	22.72	17.9
40	4087	16	12.2
41	8210	21.2	15.7
45	2300	25	18.4

Permeance of a coupon

The permeance of a given gas is calculated for each coupon using the following method. Once the coupon has been mounted and the gas has been purged, the time taken for a gas bubble to move through the burette is recorded.

$$V_{\mathrm{STP}} = V_{\mathrm{moved}}\left(\frac{T_{\mathrm{stand}}}{T_{\mathrm{actual}}} \times \frac{P_{\mathrm{stand}}}{P_{\mathrm{actual}}}\right)$$

where V_{STP} is the volume (cm^3) at standard temperature and pressure (0 °C, 1 atm), V_{moved} is the volume the gas bubble travels (cm^3), T_{stand} is the standard temperature (K), T_{actual} is the actual temperature (K), P_{stand} is the standard pressure (cmHg) and P_{actual} is the actual pressure (cmHg). Flow rate in (cm^3[STP] s^{-1}) is then calculated.

$$FR = \frac{\mathrm{V_{STP}}}{t}$$

where *FR* is the flow rate in cm^3[STP] s^{-1} and *t* is time in seconds. From here permeance (*K*) in GPU can be calculated.

$$K = \frac{1000000 \times FR}{A \times P_{\mathrm{FG}}\,(\mathrm{cmHg})}$$

where *A* is membrane area (cm^2), and P_{FG} is the pressure of the feed gas (cmHg). This is done six times for at least two different coupons. These results are averaged and can be used to calculate permeability (barrer).

$$P = K \times M_{\mathrm{thickness}}$$

where $M_{thickness}$ is the active membrane thickness (µm).

Calculating gas pair selectivity

The average permeability is used to calculate selectivity.

$$\alpha = \frac{P_a}{P_b}$$

14. References

(1) Cseri, L.; Szekely, G. Towards Cleaner PolarClean: Efficient Synthesis and Extended Applications of the Polar Aprotic Solvent Methyl 5-(Dimethylamino)-2-Methyl-5-Oxopentanoate. *Green Chem.* **2019**, *21* (15). https://doi.org/10.1039/c9gc01958h.

(2) Foster, A. B.; Tamaddondar, M.; Luque-Alled, J. M.; Harrison, W. J.; Li, Z.; Gorgojo, P.; Budd, P. M. Understanding the Topology of the Polymer of Intrinsic Microporosity PIM-1: Cyclics, Tadpoles, and Network Structures and Their Impact on Membrane Performance. *Macromolecules* **2020**, *53* (2). https://doi.org/10.1021/acs.macromol.9b02185.
